# Host-derived lipids orchestrate pulmonary γδ T cell response to provide early protection against influenza virus infection

**DOI:** 10.1038/s41467-021-22242-9

**Published:** 2021-03-26

**Authors:** Xiaohui Wang, Xiang Lin, Zihan Zheng, Bingtai Lu, Jun Wang, Andy Hee-Meng Tan, Meng Zhao, Jia Tong Loh, Sze Wai Ng, Qian Chen, Fan Xiao, Enyu Huang, King-Hung Ko, Zhong Huang, Jingyi Li, Kin-Hang Kok, Gen Lu, Xiaohui Liu, Kong-Peng Lam, Wanli Liu, Yuxia Zhang, Kwok-Yung Yuen, Tak Wah Mak, Liwei Lu

**Affiliations:** 1 Department of Pathology and Shenzhen Institute of Research and Innovation, The University of Hong Kong, Hong Kong, China; 2 Department of Microbiology, State Key Laboratory of Emerging Infectious Diseases, The University of Hong Kong, Hong Kong, China; 3 Chongqing International Institute for Immunology, Chongqing, China; 4 Department of Respiratory Medicine and Guangzhou Institute of Pediatrics, Guangzhou Women and Children’s Medical Center, Guangzhou Medical University, Guangzhou, China; 5 Bioprocessing Technology Institute, Agency for Science, Technology and Research, Singapore, Singapore; 6 Ministry of Education Key Laboratory of Protein Sciences, Center for Life Sciences, Collaborative Innovation Center for Diagnosis and Treatment of Infectious Diseases, Institute for Immunology, School of Life Sciences, Tsinghua University, Beijing, China; 7 Singapore Immunology Network, Agency for Science, Technology and Research, Singapore, Singapore; 8 Department of Pathogen Biology and Immunology, Shenzhen University School of Medicine, Shenzhen, China; 9 National Protein Science Facility, Tsinghua University, Beijing, China; 10 The Campbell Family Institute for Breast Cancer Research at Princess Margaret Cancer Centre, Ontario Cancer Institute, University Health Network, Toronto, ON Canada

**Keywords:** Interleukins, Influenza virus, Gammadelta T cells

## Abstract

Innate immunity is important for host defense by eliciting rapid anti-viral responses and bridging adaptive immunity. Here, we show that endogenous lipids released from virus-infected host cells activate lung γδ T cells to produce interleukin 17 A (IL-17A) for early protection against H1N1 influenza infection. During infection, the lung γδ T cell pool is constantly supplemented by thymic output, with recent emigrants infiltrating into the lung parenchyma and airway to acquire tissue-resident feature. Single-cell studies identify IL-17A-producing γδ T (Tγδ17) cells with a phenotype of TCRγδ^hi^CD3^hi^AQP3^hi^CXCR6^hi^ in both infected mice and patients with pneumonia. Mechanistically, host cell-released lipids during viral infection are presented by lung infiltrating CD1d^+^ B-1a cells to activate IL-17A production in γδ T cells via γδTCR-mediated IRF4-dependent transcription. Reduced IL-17A production in γδ T cells is detected in mice either lacking B-1a cells or with ablated CD1d in B cells. Our findings identify a local host-immune crosstalk and define important cellular and molecular mediators for early innate defense against lung viral infection.

## Abstract (web summary)

Influenza A infection results in γδ T cell influx and production of IL-17 in the lungs. Here, the authors show that this effect is primed by CD1-restricted ligands that are released by infected cells and presented by B1a cells in the lungs.

## Introduction

Influenza A virus is a major causative pathogen of acute respiratory diseases worldwide and causes substantial morbidity and mortality in infected patients. An appropriate induction of immune responses in the lung is necessary for virus elimination and host recovery. The pulmonary immune system harbors a unique environment gathering a group of special cell subsets and molecular components, which are necessary for the maintenance of immunological homeostasis and pathogen clearance in the lung^1^. Recent studies with systems-level analysis indicate that a series of host components such as lipids and metabolites can modulate the proinflammatory response of lung epithelial cells against either 1918 or 2009 human pandemic H1N1 (pdmH1N1) influenza viruses^2^. In addition, the ratio of specific species of lipids was found to be markers of influenza virus pathogenicity in mice^3^. However, it is unclear how lipids regulate early immune protection against influenza infection.

Various innate and adaptive immune components are activated to mediate the clearance of respiratory viral infections, especially those with rapid kinetics of antiviral responses during primary infection. In both humans and mice, γδ T cells represent a major T cell population in the barrier sites, suggesting their crucial involvement in barrier surveillance and first-line defense^4^. This preferential distribution of γδ T cells favors their “kick-starting” activities^5^ in driving immunity against invasive pathogens in situ by secreting proinflammatory cytokines including IL-17A^5,6^, IFN-γ^7^, M-CSF^8^, IL-9^9^, or Amphiregulin^10^ upon activation. Mounting evidence indicates that both human and mouse γδ T cells exert protective function in control infections by different strains of the influenza virus, such as seasonal H1N1, H5N1, and H9N2 viruses^7,11–14^. Moreover, drug-induced expansion of human Vγ9Vδ2 T cells is critically involved in elimination of influenza virus in humanized mice^15^. We have also found that γδ T cells rapidly infiltrate into lung tissue and produce IL-17A in response to H1N1 (A/PR/8/34) infection^6,16^. However, it is largely unclear how γδ T cells are rapidly activated in the lung during influenza infection as well as the underlying mechanisms. Moreover, the cellular heterogenicity and effector function of lung γδ T cells against influenza viruses remain poorly characterized.

Available evidence on the multifaceted contributions of γδ T cells indicates that γδ T cells comprise subpopulations and readily differentiate under pathological settings^4,17^. Although hemagglutinin (HA) from H5N1, but not H1N1, influenza is found to directly trigger γδ T cells to produce IFN-γ by binding to sialic acid receptors on the cells^7^, many γδ T cells utilize a relatively limited repertoire to recognize nonpeptide antigens and stress-induced ligands presented upon the major-histocompatibility-complex (MHC)-class-I-like molecule CD1d^18,19^. Moreover, productive T cell antigen receptor (TCR)-ligand interactions are prone to trigger microbiota associated-IL-17A production in γδ T cells^20,21^. Up to date, it remains obscure how influenza viruses activate CD1d-dependent T cells despite an apparent lack of virus-derived CD1d ligands^22^.

Increasing evidence indicates a critical role of innate B-1 cells in antiviral immune responses. We have shown that CD1d-expressing B-1a cells, a subset of innate B-1 cells, rapidly infiltrate into the lung tissue and secrete natural antibodies, paralleling to early IL-17A production during influenza infection^6^. We and others have also found that autoreactive B cells can present endogenous lipid antigens^23^ or glycolipid antigens on the surface of *Sphingomonas species*, *Borrelia burgdorferi*, and *Streptococcus pneumoniae*^24,25^, to activate NKT cells in a CD1d-dependent manner^22,24^. Thus, it remains to be established how these infiltrated B-1 cells interact with other immune cells during early innate response. Therefore, further identification of early activated immune cells in the lung parenchyma and underlying molecular mechanisms in host defense is vital to developing effective treatment strategies.

In this study, we provide in vivo evidence of an early pulmonary immune response against pdmH1N1 influenza virus dominated by Tγδ17 cells starting from day 1 of respiratory exposure in mice. Single-cell RNA sequencing (scRNA-seq) analysis of lung-infiltrating γδ T cells revealed that respiratory influenza virus infection-induced activation of TCRγδ^hi^CD3^hi^AQP3^hi^CXCR6^hi^ cells with a dominant IL-17A signature, a population maintained by thymic output and acquires tissue-resident feature after pulmonary imprinting. Moreover, lung-infiltrating B-1a cells present lung tissue-derived lipid antigens to γδ T cells and elicit IL-17A production by γδ T cells via IRF4. Importantly, complementary analysis on pneumonia patients identified positive associations between Vγ9Vδ2 cells, accumulation of cardiolipin (CL) lipids in respiratory tract and disease severity. Together, our results reveal both cellular and molecular mechanisms of local host-immune crosstalk occurring immediately after infection that orchestrates innate B–T interaction for early defense against influenza virus.

## Results

### PdmH1N1 infection induces γδ T cell infiltration into lung tissue

**Fig. 1:**
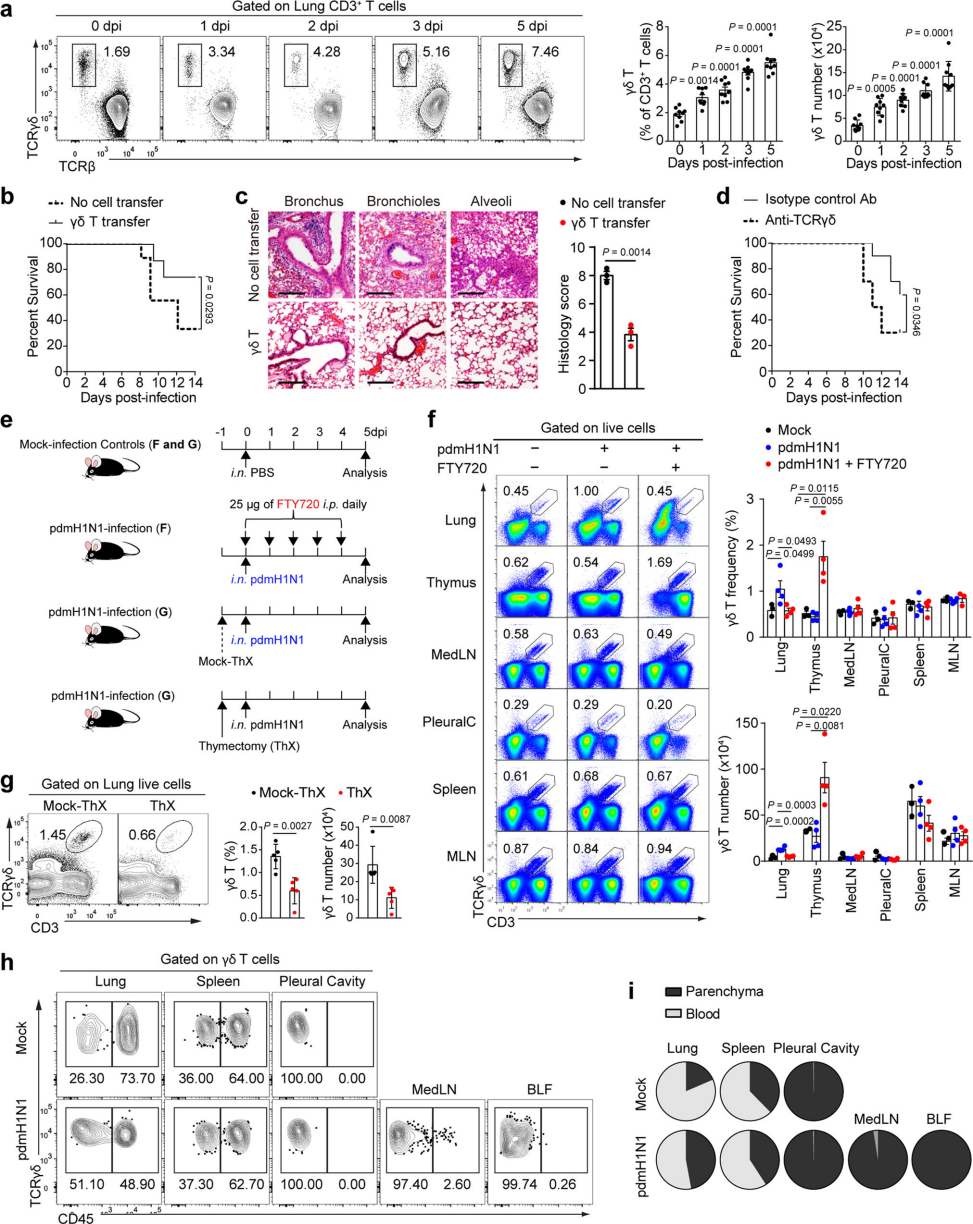
PdmH1N1 infection induces infiltration of thymus-derived γδ T cells into lung. **a** Representative plots (left) and frequency and number (right) of γδ T cells in lungs of pdmH1N1-infected mice (*n* = 9). **b, c** Mice at 1 dpi were i.v. transferred with or without 2 × 10^6^ γδ T cells purified from mice at 4 dpi. **b** Kaplan–Meier survival rate (*n* = 9, 10). **c** Representative hematoxylin and eosin (H&E) histology (left) and scoring (right) of lungs from mice at 5 dpi (*n* = 3). Images are at original magnification ×200. Scale bar, 40 μm. **d** Kaplan–Meier survival rate of mice with i.p. injected 200 μg of anti-TCRγδ or control antibodies 3 days before infection (*n* = 10). **e** Schema of FTY720 injection (**f**) and thymectomy (ThX) surgery (**g**). **f** Flow cytometry plots (left) and cumulative data (right) showing frequencies and numbers of CD3^+^TCRγδ^+^ cells in gated live singlets from the indicated organs (*n* = 3, 4, 4). MedLN mediastinal lymph nodes, PleuralC pleural cavity, MLN mesenteric lymph nodes. **g** Mice received ThX or mock surgery 1 day before infection (*n* = 5). Representative plots (left) and cumulative data (right) showing the frequencies and numbers of CD3^+^TCRγδ^+^ cells from lungs at 4 dpi. **h, i** Mock-infected and pdmH1N1-infected mice at 5 dpi were i.v. injected with 40 μg of FITC-conjugated antibody to CD45 10 min before they were killed. Mice were perfused with PBS and tissues were harvested and analysed. Since MedLN is only detectable from 2 dpi onwards, and γδ T cells are only detectable in BLF of infected mice, analysis of γδ T cells in MedLN and BLF was conducted only with infected mice. Representative plots (**h**) and frequency pie charts (**i**) showing circulating γδ T (CD45^+^) or parenchyma-associated γδ T cells (CD45^−^) in gated CD3^+^TCRγ^δ+^ cells (*n* = 5). Data are combined from two or three independent experiments and presented as mean ± SEM. *P* values were determined using two-tailed unpaired Student’s *t*-test (**c, g, h**), Gehan-Breslow-Wilcoxon Test (**b, d**) or one-way ANOVA (**a, f**). Source data are included in Source Data file.

To investigate γδ T cell response to lung viral infection, we infected C57BL/6 mice with pdmH1N1 influenza virus. As early as 1 day post-infection (dpi), we observed a significant increase in both frequency and number of γδ T cells (Fig. 1a), whereas the accumulation of other innate responding immune cells such as NK and NKT cells were only increased from 3 dpi onwards (Supplementary Fig. 1a). Intravenously (i.v.) transferred γδ T cells significantly increased the survival rate and markedly reduced lung pathology of lethally infected mice (Fig. 1b, c). On the contrary, targeting γδ T cells in vivo using an anti-TCRγδ monoclonal antibody (mAb, clone GL3)^8,26^ before onset of the infection resulted in significantly reduced survival of mice compared with isotype control antibody-treated mice (Fig. 1d). These data indicated that γδ T cells play a protective role against pdmH1N1 infection in mice.

γδ T cells constitute the major subset of resident T cells that is closely aligned with innate immunity in mucosa barriers like skin^4^. However, their exchange between tissues and lung-specific adaptation remains poorly understood. To examine whether tissue recruitment contributes to lung γδ T cell pool after infection, we treated mice with FTY720, a sphingosine 1 phosphate (S1P) receptor agonist that blocks egress of lymphocytes from lymphoid organs and stops cell trafficking from circulation into lung tissues (Fig. 1e, f). After FTY720 treatment, the frequency and number of lung γδ T cells in virus-infected mice were considerably reduced while those of thymic counterparts were dramatically increased. In contrast, the frequencies and numbers of γδ T cells in mediastinal lymph node (MedLN), pleural cavity, spleen, and mesenteric lymph nodes (MLN) remained unchanged (Fig. 1f). Importantly, thymectomy in mice 24 h before viral infection substantially reduced the frequencies and total numbers of γδ T cells at 4 dpi (Fig. 1e, g), providing strong evidence that the maintenance of lung γδ T cell population during influenza infection is highly dependent on new migrants from the thymus.

The lungs are highly vascularized, and a large proportion of immune cells are actually circulating cells trapped in the capillary beds that surround the air sacs^27^. To examine the localization of lung γδ T cells within distinct anatomical niches, namely the vasculature, airway and interstitial spaces, we i.v. injected fluorescein isothiocyanate (FITC)-labeled mAb against CD45 into mock- or influenza-infected mice and assessed its binding to γδ T cells as a criterion to discriminate circulating cells from parenchyma-bound cells. Single-cell suspensions from various organs of PBS-perfused mice were stained for TCRγδ and analysed by flow cytometry. γδ T cells were detected in bronchoalveolar lavage fluid (BLF) of infected but not naïve mice, suggesting entry of γδ T cells into the bronchoalveolar space upon infection (Fig. 1h). We assessed CD45^+^ cells proceeding from the vasculature and CD45^−^ cells residing in the parenchyma of various organs. Following infection, the proportion of CD45^−^ γδ T cells in the parenchyma among total γδ T cells in the lung markedly increased. In contrast, the proportions of CD45^+^ and CD45^−^ γδ T cells remained largely unchanged in the spleen and pleural cavity, and only very small proportions of γδ T cells in MedLN and BLF stained positive for anti-CD45 mAb (Fig. 1h, i). These data suggest that the majority of γδ T cells in lungs originally circulate in the blood during steady state and rapidly migrate into the lung parenchyma and then into respiratory tract upon viral infection.

### Lung γδ T cells protect against pdmH1N1 infection by producing IL-17A

**Fig. 2:**
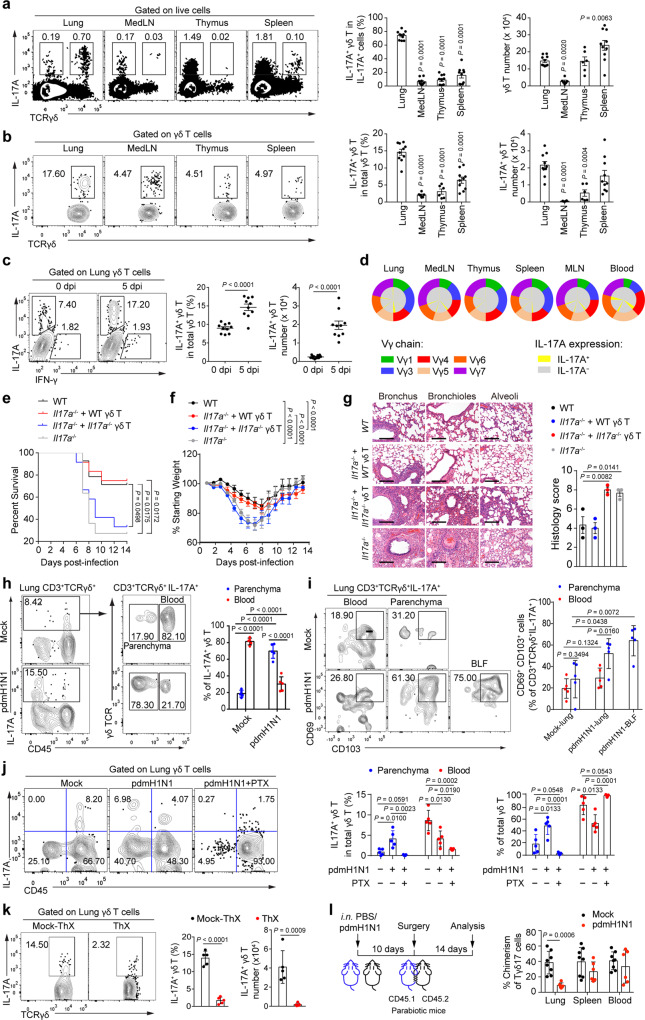
Lung γδ T cells protect against pdmH1N1 by producing IL-17A. **a** Representative plots showing IL-17A^+^ cells (left) and cumulative data (right) showing frequencies of IL-17A^+^ γδ T cells in IL-17A^+^ population (*n* = 10) and cell numbers of γδ T cells (*n* = 10, 6, 6, 10) at 5 dpi. **b** Plots (left) and cumulative data (right) showing IL-17A^+^ γδ T cells at 5 dpi (*n* = 10, 6, 6, 10). **c** Plots (left) and cumulative data (right) showing IL-17A^+^ γδ T cells at 5 dpi (*n* = 10). **d** Flow cytometry analysis of Vγ chain usage and IL-17A expression by γδ T cell subtypes (*n* = 6). **e–g** PdmH1N1-infected *Il17a*^−/−^ mice at 1 dpi were i.v. transferred with 2 × 10^6^ γδ T cells purified from infected wild-type (WT) or *Il17a*^−/−^ mice at 4 dpi. Kaplan–Meier survival rate (**e**) and body weight (**f**) were monitored (*n* = 14, 12, 12, 11). **g** H&E histology (left) and scoring (right) of lungs at 5 dpi (*n* = 3). Scale bar, 40 μm. **h, i** Mice at 5 dpi were i.v. injected with 40 μg of FITC-anti-CD45 antibody 10 min before killed and perfused (*n* = 5). **h** Plots (left) and cumulative data (right) showing lung circulating (CD45^+^) and parenchyma-associated (CD45^−^) IL-17A^+^ γδ T cells. **i** Plots (left) and cumulative data (right) showing frequencies of CD69^+^CD103^+^ cells. **j** Mice received an i.p. injection of 1 μg of PTX immediately after infection. At 4 dpi, mice were injected with FITC-anti-CD45 10 min before they were killed and analyzed (*n* = 5). **k** Mice received ThX or Mock-ThX 1 day before infection (*n* = 5). Plots (left) and cumulative data (right) showing IL-17A^+^ γδ T cells at 4 dpi. **l** Schema of parabiosis (left) and chimerism of Tγδ17 cells (right) (*n* = 8, 6). Data are combined from two or three independent experiments and presented as mean ± SEM. *P* values were determined using two-tailed unpaired Student’s *t*-test (**c, h–l**), Gehan-Breslow-Wilcoxon Test (**e**), one-way ANOVA (**a, b, g**), or two-way ANOVA (**f**). Source data are included in Source Data file.

When we analysed the cytokine profile and other effector molecules expressed by lung-infiltrating γδ T cells by flow cytometry, we found the cells predominantly expressed IL-17A and barely other cytokines such as IL-10, IL-4, IL-6, IL21, and IFN-γ, indicating that IL-17A is a signature cytokine of lung γδ T cells upon influenza infection (Supplementary Fig. 1b). Since our previous studies showed that IL-17A protects against influenza infection^6,16^, we next asked if γδ T cells mediate such protection by producing IL-17A. We found that the frequency of lung Tγδ17 cells among total IL-17A-producing cells was significantly higher than those of counterparts in MedLN, thymus and spleen (Fig. 2a). The frequency of IL-17A-producing cells within the γδ T cell population was also significantly higher in the lung than those of counterparts in other organs examined (Fig. 2b), suggesting γδ T cells are the major source of IL-17A in the lung at early stage of infection. Moreover, pdmH1N1 infection induced a significant increase of IL-17A production in lung-infiltrating γδ T cells (Fig. 2c). Lung γδ T cells also exhibited a Vγ chain usage pattern, which was distinct from those utilized by γδ T cells present in other organs (Fig. 2d). Consistent with an important role for IL-17A in protection against influenza infection, pdmH1N1-infected IL-17A-deficient (*Il17a*^−/−^) mice showed significantly reduced survival rate, increased weight loss and lung injury when compared with infected wild-type mice. Furthermore, transfer of wild-type but not *Il17a*^−/−^ γδ T cells markedly rescued *ll17a*^−/−^ mice from death (Fig. 2e–g). Together, these data suggest that IL-17A produced by γδ T cells is required to protect against pdmH1N1 infection in mice.

We next investigated the phenotypic feature and differentiation state of lung Tγδ17 cells. Compared to their IL-17A^−^ counterparts, lung Tγδ17 cells expressed high levels of CD25, CD69, NKG2D, CD44, CD24, PD-1, IL-7R, and CD62L, and a low level of NKG2A, indicating a more active state of lung Tγδ17 cells. In addition, Tγδ17 cells also expressed low levels of NK1.1, CD40, and CD27, which are known as fate determinants of functional diversities responsible for cytotoxicity, antigen presentation and IFN-γ expression, respectively^28^ (Supplementary Fig. 1c). To further investigate whether the unique microenvironments influence Tγδ17 cell biology, we compared the expression of canonical markers CD11a, CD69, and CD103 associated with tissue retention of CD4^+^ and CD8^+^ T cells on Tγδ17 cells in different compartments of the lung^29–31^. By i.v. injecting FITC-labeled mAb against CD45, flow cytometric analysis detected markedly increased frequencies of Tγδ17 cells with respect to total γδ T cells in the parenchyma of infected lungs (Fig. 2h). CD11a was found highly expressed but comparable in Tγδ17 cells in blood and lung tissues (Supplementary Fig. 1d). Intermediate levels of CD69 and CD103 expression were detected on Tγδ17 cells from the MedLN, spleen and pleural cavity but much higher levels of CD69 and CD103 expression were found on their counterparts in the lung and BLF (Supplementary Fig. 1e). Upon infection, frequencies of CD69^+^CD103^+^ Tγδ17 cells with respect to total Tγδ17 cells significantly increased in the parenchyma of lung and BLF with the highest frequencies in the latter. The frequency of these cells in the vasculature was, however, unaffected (Fig. 2i). Additionally, we found that a short pretreatment of pertussis toxin (PTX), an inhibitor of chemokine receptor signaling^32^, prevented the migration of γδ T cells into the lung parenchyma (Fig. 2j). Consistent with a reduction of lung γδ T cells in mice removed thymus, thymectomy surgery substantially reduced the frequency and number of lung Tγδ17 at 4 dpi (Fig. 2k). Using a parabiosis approach with CD45.1 and CD45.2 congenic mice, the conjoined mice developed expected high levels of chimerism in circulating and lung Tγδ17 cells before viral infection. However, there was minimal chimerism in lung Tγδ17 cells in the respective parabionts following infection (Fig. 2l). Collectively, these results suggest that at the early stage of influenza infection, thymic output via circulation continually sustains the γδ T cell pool in the lung parenchyma and airway. Following trafficking, these cells progressively adapt to the microenvironment and acquire tissue-resident features.

### Lung Tγδ17 cells exhibit distinct transcriptome of functional maturity

**Fig. 3:**
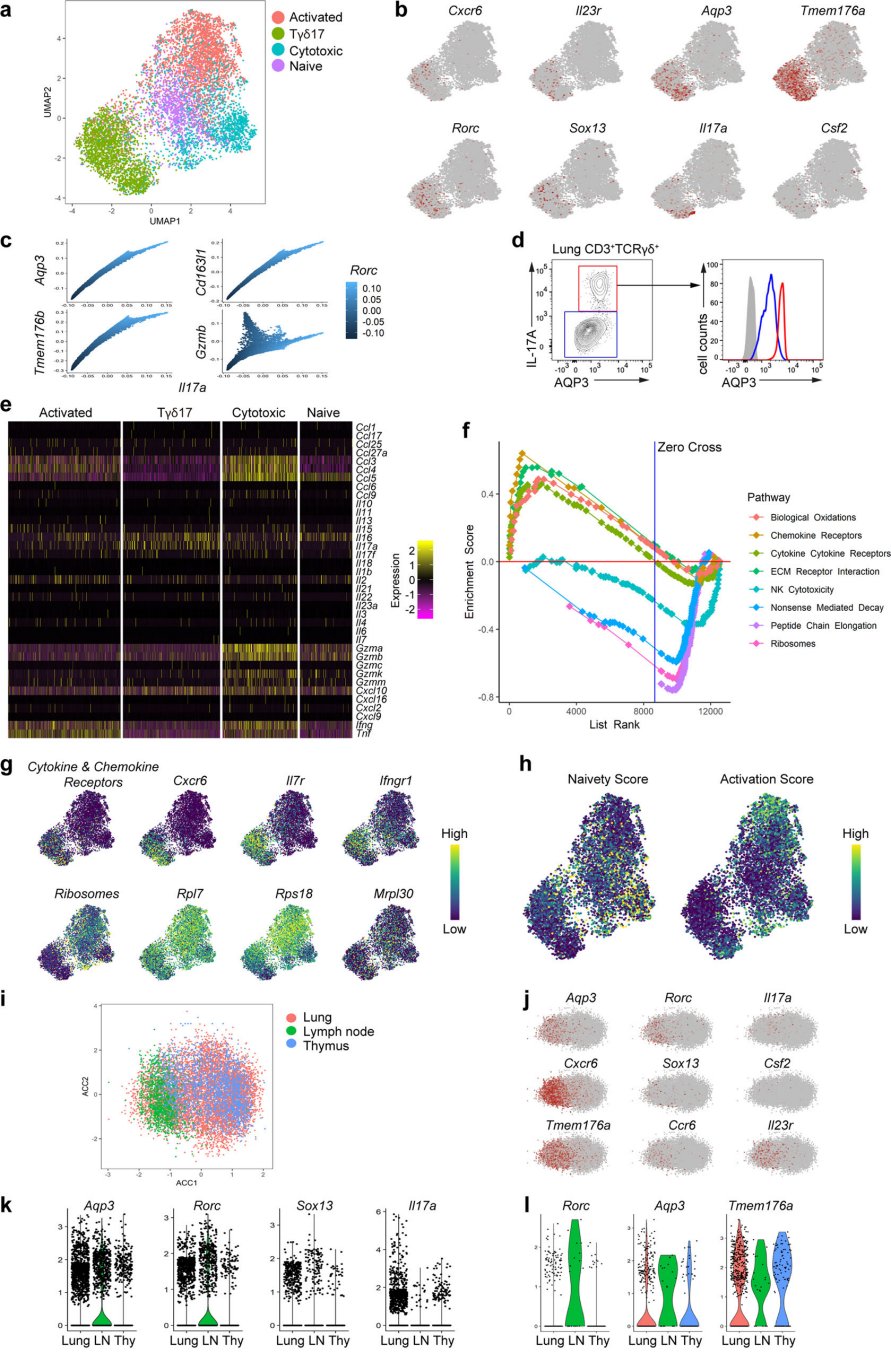
Lung Tγδ17 cells have a distinct transcriptome indicative of functional maturity. **a** Sorting-purified single γδ T cells from lungs at 4 dpi were subjected to scRNA-seq. UMAP clustering following dimension reduction based on highly variable genes across the 7863 single cells recovered revealed the formation of 4 primary clusters of distinct cell types that were then assigned identifiers based on their expression profiles. **b, c** Visualization of the expression profiles for key genes previously reported to be associated with Tγδ17 in **a**. **d** Representative flow cytometric plots showing expression of AQP3 and IL-17A in gated CD3^+^TCRγδ^+^ cells from pdmH1N1-infected lungs at 4 dpi. **e** Heatmap visualization of the expression patterns of the most varied genes in each cluster across each cell in the cluster. These highly varied genes were used to help annotate the clusters. **f** Representative GSEA results generated using distinct gene list databases (KEGG, Hallmark, Reactome) as visualized through UMAP confirmed that the substantial heterogeneity observed between the γδ T clusters was driven by significant differences in gene expression along unique biological pathways/processes. Eight significantly variant pathways between the activated and Tγδ17 clusters are shown here as these 2 clusters are highly separated within the UMAP space and in GSEA analysis. **g, h** UMAP plots show the expression profiles of genes associated with Tγδ17 in **a**. **i** Correlation analysis of scRNA-seq data with previously published sequencing of Tγδ17 cells (GSE123400) as shown visually through UMAP following canonical correlation analysis. **j** UMAP visualization of the expression levels of prominent genes associated with Tγδ17 cells. **k, l** Violin plots of the expression profiles of markers associated with Tγδ17 in **i**.

To characterize the molecular features of lung γδ T population during influenza infection, we purified CD19^−^CD3^+^TCRγδ^+^ cells from lung tissues at 4 dpi for scRNA-seq analysis. After initial quality control procedures, 7863 single cells expressing a median of 1043 genes passed the threshold and were used for clustering analysis via Uniform Manifold Approximation and Projection (UMAP) (Supplementary Fig. 2a). Four primary clusters of distinct cell types were recovered from this analysis and were assigned identifiers based on their expression of known marker genes, including activated, Tγδ17, cytotoxic, and naïve clusters (Fig. 3a and Supplementary Fig. 2b). While some differences in the expression of classical cell cycle (*Pcna, Hmgb2*) or apoptosis (*Bax, Casp3*)-related genes were observed among the clusters, these were not key drivers of cluster formation (Supplementary Fig. 2c). Instead, direct comparison of the clusters showed that Tγδ17 cells were highly enriched for 22 genes in terms of both expression and detection rate, and a significant proportion of the cells positive for each marker were also *Il17a*^*hi*^ (Supplementary Fig. 2d). While known transcription factors (*Rorc*, *Sox13*) and surface markers (*Ccr6, Il23r*) associated with the known phenotype of Tγδ17 cells, other less well-characterized markers such as *Aqp3*, and *Tmem176a* were actually more prominently identified (Fig. 3b). Since droplet-based scRNA-seq data are highly prone to technical dropout, we then utilized a graph-based imputation method to clarify the potential relations between them. Consistent with our expectations, the imputed data revealed strong correlations in the expression profiles of the markers we identified with both *Il17a* and *Rorc*, while showing no relationship with the unrelated cluster marker *Gzmb* (Fig. 3c). In order to validate that these markers are indeed robust, we then used flow cytometry to clarify the correlations on a protein level, wherein we observed a clear relationship between AQP3 expression and IL-17A^+^ status (Fig. 3d), establishing it as a useful phenotype marker.

Beyond this preliminary marker analysis, we also explored possible functional differences between the clusters. Heatmap visualization of the differentially expressed genes between the clusters demonstrated that the Tγδ17 cluster had much less prominent expression of several chemokines, interferon, and cytotoxic factors (Fig. 3e). However, representative geneset enrichment analysis (GSEA) results derived from averaged bulk expression profiles of each cluster demonstrated Tγδ17 cells had enhanced expression of a number of cytokine and chemokine receptors and also displayed enrichment for mitochondrial respiratory capacity (Fig. 3f, and Supplementary Fig. 2e, f). Indeed, the elevated expression of these cytokine receptors and decreased ribosomal RNA levels relative to the other clusters (Fig. 3g) is consistent with a recognized role of ribosomal capacity during T cell functional maturation^33^, a trend also observable via independent assessment of each cell for markers of T cell naivety or activation (Fig. 3h).

In order to explore the generality of the cell phenotype observed, we also further performed paired analysis of our data with existing scRNA-seq results on γδ T cells (GSE123400)^34^. Canonical correlational analysis between the sets demonstrated that the two datasets could fit reasonably well together, with a region of the combined data including Tγδ17 cells (Fig. 3i, j). Violin plots confirmed that lung Tγδ17 cell cluster was closely aligned to their counterparts in thymus and lymph nodes (Fig. 3k). Notably, this subset included Tγδ17 cells from all three origins, and these latter cells featured high expression of *Aqp3*, suggesting that it was not a lung-specific marker, but more broadly associated with the Tγδ17 phenotype (Fig. 3l). As MedLN is only clearly visible after 2 dpi, these findings suggest γδ T cells may undergo differentiation after emigrating from thymus. Overall, lung Tγδ17 cells represent a distinct γδ T cell subtype with a unique transcriptome reflective of functional maturity.

### The γδTCR-IRF4 axis regulates IL-17A production in lung γδ T cells

**Fig. 4:**
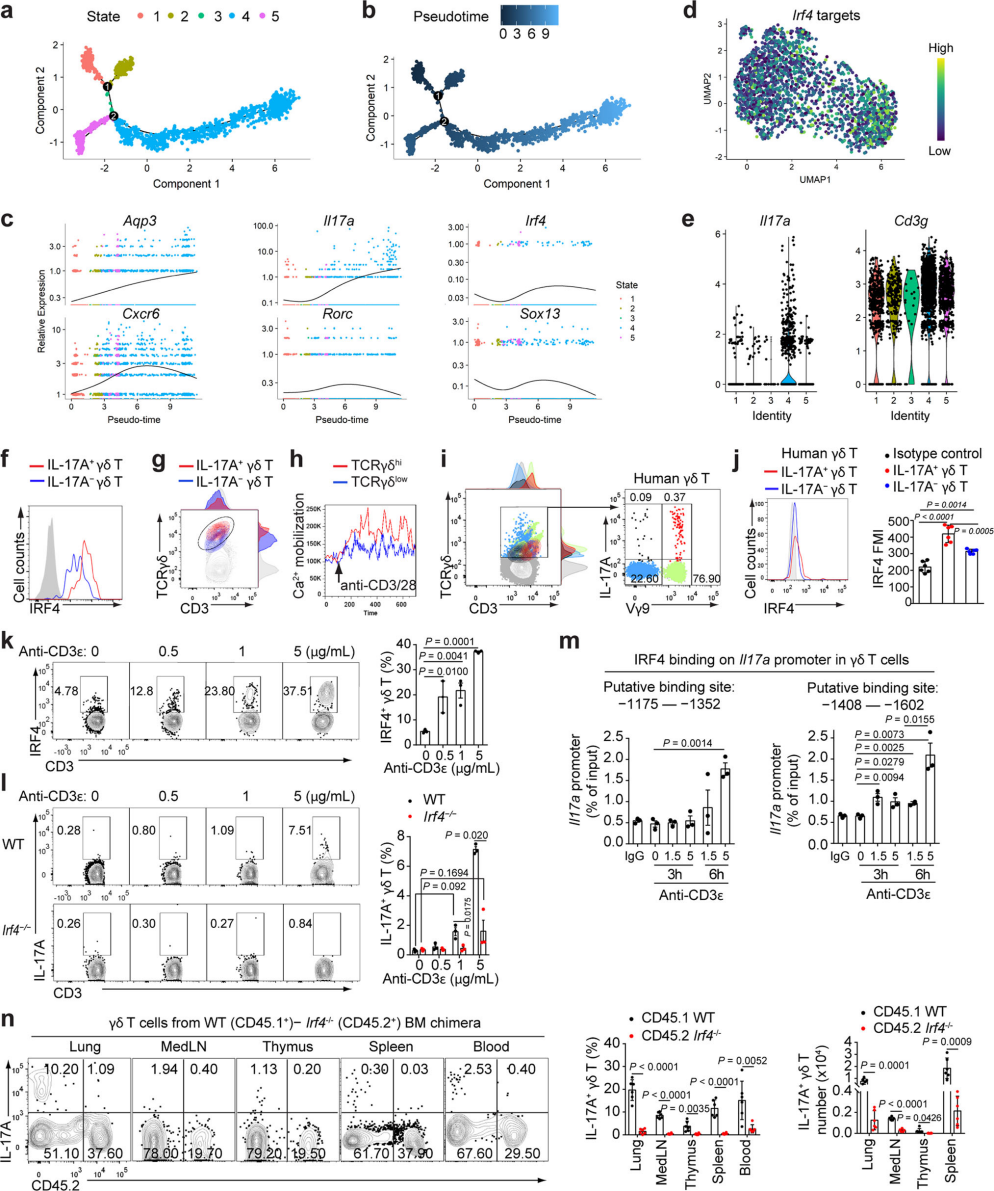
The γδTCR-IRF4 axis regulates IL-17A production in lung γδ T cells. **a, b** Trajectory map generated using DDTree reduction as implemented through Monocle on a list of 1224 genes with detectable expression and significant dispersion within the Tγδ17 subset. **c** Pseudotime mapping of gene expression levels within the Tγδ17 subset. **d** UMAP visualization of IRF4 targeted genes that enrich in the Tγδ17 subset. **e** Violin plots of the gene expression of *Il17a* and *Cd3g* separated by the projected cell state demonstrates that state 4, which has progressed the furthest along pseudotime, is composed of cells that highly express those 2 genes. **f** IRF4 expression in γδ T cells in mouse lungs at 5 dpi. Shaded histograms depict staining by isotype control antibody. **g** Representative plot with adjunct histograms gated on lung CD3^+^ live singlets at 5 dpi. **h** Mouse γδ T cells cells (2 × 10^6^/mL) were stimulated with soluble anti-CD3/CD28 beads (5 μg/mL). Kinetic diagrams showing the merged calcium mobilization in TCRγδ^hi^ and TCRγδ^low^ cells. **i** Plots with adjunct histograms gated on CD3^+^ (left) or CD3^+^ TCRγδ^+^ (right) live singlets of human PBMCs. **j** IRF4 expression in γδ T cells from human PBMCs. Mean fluorescent intensity, MFI (*n* = 6). **k, l** Expression of IRF4 (**k**) and IL-17A (**l**) on day 3 of purified γδ T cells from WT or *Irf4*^−/−^ mice cultured with plate-bound 1 μg/mL of anti-CD28 and various concentrations of anti-CD3ε (*n* = 3). **m** γδ T cells were stimulated with 1 μg/mL of anti-CD28 and 1.5 or 5 μg/mL of anti-CD3ε. ChIP quantification of IRF4 binding to the *Il17a* promoter was performed using quantitative PCR (*n* = 3). **n** IL-17A production in WT and *Irf4*^−/−^ bone marrow chimera were analysed at 5 dpi (*n* = 6). Data are combined from two or three independent experiments and represented as mean ± SEM. *P*-values were determined using two-tailed unpaired Student’s *t*-test (**j, l, m, n**) or one-way ANOVA (**k**). Source data are included in Source Data file.

To understand the development path followed by lung γδ T cells during infection, we next performed trajectory analysis of the Tγδ17 cells identified in our scRNA-seq data. Trajectory and pseudotime mapping of the subset showed that two distinct progenitor states and two end states were followed by the cells, with progression through a joint intermediate state (Fig. 4a, b). Differential gene expression analysis revealed that expression of the inhibitory transcription factor *Tcf7* and STAT-signaling inhibitor *Socs3* both decreased as a function of the pseudotime, confirming correctness of the time direction since downregulation of above genes with functional maturity of T cells and hence pseudotime is expected (Supplementary Fig. 3a). While *Il17a* expression monotonically increased over pseudotime, *Aqp3* displayed an almost linear correlation with pseudotime progression, suggesting that it may be able to predict the developmental maturation of IL-17A-secreting cells (Fig. 4c and Supplementary Fig. 3b). On a regulatory level, *Irf4* was more highly expressed in both of the end states in a similar fashion as *Il17a*, and exhibited a transient induction among many of the common transcription factors reported to be involved in IL-17A production^35^. Furthermore, the total expression levels of its known target genes also exhibited a trend towards being increased in *Il17a*^*hi*^ cells, with this trend being observable following independent pseudotime algorithms (Fig. 4d and Supplementary Fig. 3c–e). Interestingly, these *Il17a*^*hi*^ cells exhibit elevated expression of *Cd3g* (Fig. 4e).

Consistent with preceding correction data, flow cytometric analysis confirmed that lung Tγδ17 cells expressed higher protein levels of IRF4 (Fig. 4f). These cells also displayed elevated levels of TCRγδ and CD3 than their IL-17A^−^ counterparts (Fig. 4g). Intracellular calcium flux in γδ T cells after stimulation with anti-CD3ε and anti-CD28 mAbs also showed a close correlation between the signaling intensity and the levels of TCRγδ expression, suggesting an association between the expression level and signal strength of TCRγδ (Fig. 4h). Remarkably, Tγδ17 cells in human peripheral blood mononuclear cells (PBMCs) exhibited similar TCRγδ^hi^CD3^hi^ phenotype and were enriched in the Vγ9^+^ population (Fig. 4i). Notably, IL-17A expression was also correlated with higher IRF4 expression in human γδ T cells, displaying similar molecular features of human Tγδ17 cells to murine counterparts (Fig. 4j). Both antigen dose and TCR signal strength were previously shown to influence the thymocyte fate and the effector differentiation of T cells^36,37^, possibly by regulating the expression intensity of key transcription factors^38^. Previous findings that IRF4 is a crucial transcription factor for sensing the intensity of TCR receptor signaling to scale the expression amount of itself and coordinate CD4^+^ T cell polarization^38,39^ prompted us to investigate whether IRF4 mediated effector differentiation of γδ T cells. Indeed, IRF4 was induced by anti-CD3ε mAb in a concentration-dependent manner stimulating increasing TCR signal strength (Fig. 4k, Supplementary Fig. 4a, b). IL-17A expression also increased upon TCR signaling intensity in a similar dose-dependent fashion (Fig. 4l). Notably, IRF4 deficiency in γδ T cells completely abrogated IL-17A induction by anti-CD3ε, suggesting that IRF4 is required for γδTCR ligation-induced IL-17A production in γδ T cells (Fig. 4l). Interestingly, IRF4 was induced upon weak TCR stimulation (0.5 μg/mL of anti-CD3ε) whereas IL-17A was induced upon stronger TCR stimulation (1 μg/mL of anti-CD3ε) (Fig. 4k, l), suggesting the magnitude of IRF4 expression is highly sensitive to TCR signal strength while a certain TCR signal threshold is required to initiate IL-17A production. Moreover, chromatin immunoprecipitation (CHIP) analysis demonstrated that IRF4 bound directly to the promoter of *Il17a* gene in a manner dependent on TCR signal strength. Binding of IRF4 to the −1175 to −1352 locus of the *Il17a* gene promoter was only observed at high TCR signal strength (5 μg/mL of anti-CD3ε). However, the binding of IRF4 at the −1408 to −1602 locus was achieved at lower TCR signal strength (1.5 μg/mL of anti-CD3ε) (Fig. 4m), suggesting that this locus contains high-affinity IRF4-binding sites. Thus, abundance of IRF4 expression determined by strength of TCR signaling is key to regulating *Il17a* transcription in γδ T cells. In addition, because IRF4-binding sites on the *Il17a* promoter varied in affinity for the transcription factor, the site binding of IRF4 with low affinity requires stronger TCR ligation. Taken together, these data reveal a crucial requirement of TCR-mediated signaling for IL-17A induction in γδ T cells.

To determine whether IRF4 affects IL-17A expression and hence survival from influenza infection in vivo, IRF4-heterozygous (*Irf4*^+/−^) and deficient (*Irf4*^−/−^) mice were infected with pdmH1N1 virus. *Irf4*^−/−^ mice exhibited significantly reduced survival and increased body weight loss and developed more severe lung pathology compared with *Irf4*^+/−^ controls (Supplementary Fig. 4c–e), suggesting an important role for IRF4 in protective immunity against pdmH1N1 infection. Further characterization of pdmH1N1-infected *Irf4*^−/−^ mice revealed significantly reduced IL-17A production by γδ T cells in the lung, MedLN, thymus, spleen and blood compared with *Irf4*^+/−^ mice (Supplementary Fig. 4f). Next, we generated chimeras by reconstituting lethally irradiated wild-type (CD45.1^+^) recipient mice with congenitally marked bone marrow cells from *Irf4*^−/−^ (CD45.2^+^) donor mice and wild-type (CD45.1^+^) donor mice at a 1:1 ratio. At 5 dpi, γδ T cells of *Irf4*^−/−^ origin exhibited a significantly lower frequency of IL-17A^+^ population than those of wild-type origin (Fig. 4n), indicating a T cell-intrinsic function for IRF4 in promoting Tγδ17 cell differentiation.

To rule out the possibility that the impaired Tγδ17 differentiation of *Irf4*^−/−^ γδ T cells was due to aberrant development of γδ T cells, we characterized Tγδ17 subsets in the chimeras. Although the frequency of γδ T subsets of *Irf4*^−/−^ origin was consistently lower than that of wild-type origin, we did not observe an appreciable difference between their composition of Vγ chain usage (Supplementary Fig. 4g). Moreover, the frequency of IL-17A^+^ γδ T cells of *Irf4*^−/−^ origin was significantly lower than that of wild-type origin (Supplementary Fig. 4h). Overall, these data demonstrate that γδTCR-IRF4 pathway is indispensable for early IL-17A production in lung γδ T cells during influenza virus infection.

### Infection-induced endogenous lipids promote γδ T cell IL-17A production

**Fig. 5:**
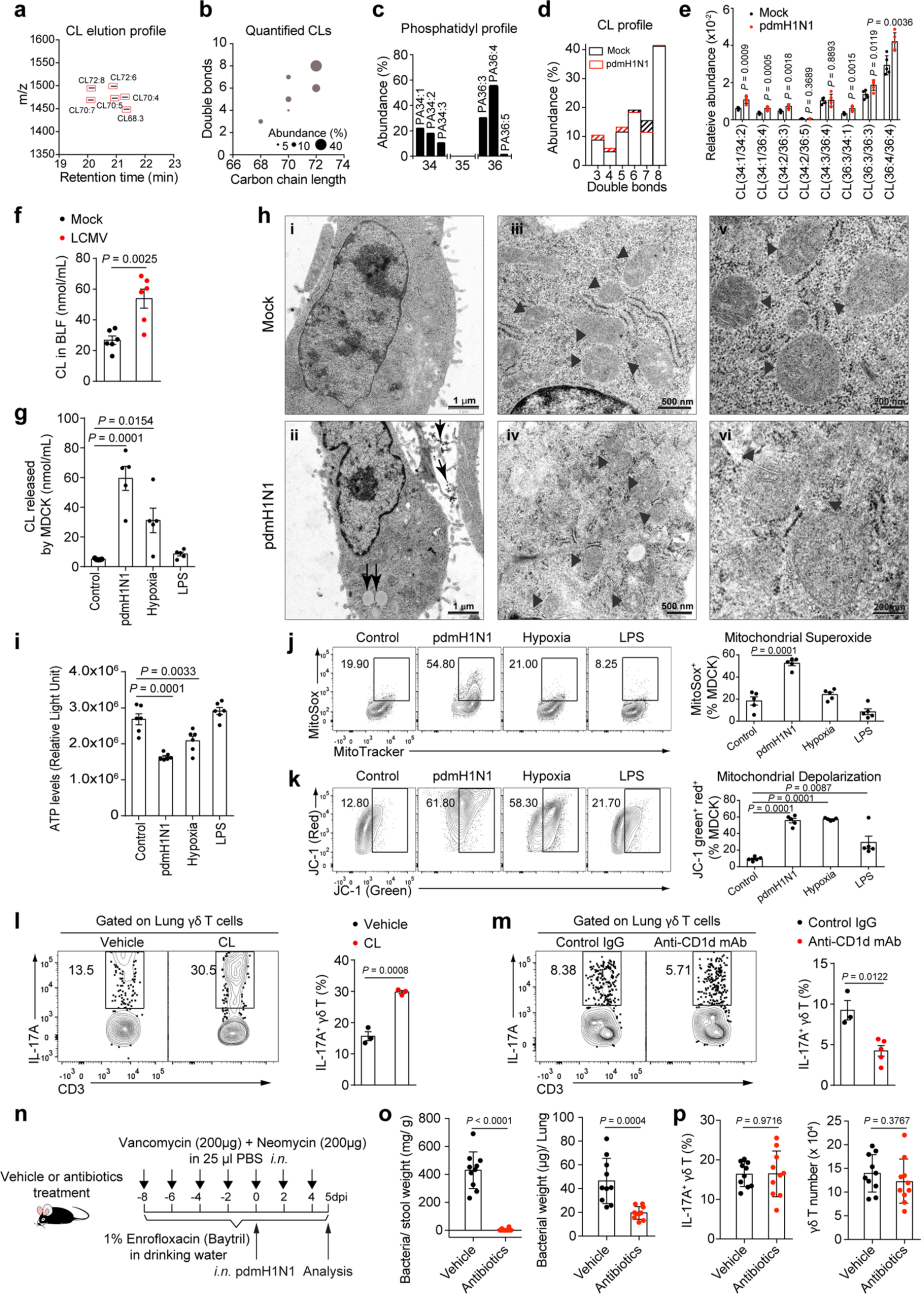
Infection-induced endogenous lipids promote γδ T IL-17A production. **a–e** LC-MS/MS assessment of CL species in BLF from infected mice at 5 dpi (*n* = 5). **a** CL elution profile. **b** Abundance of identified CLs with various carbon chain length and double-bond components. Circle sizes correspond to abundance of chromatographic peak areas of CLs among identified CL species. **c** Phosphatidyl (PA) composition in CLs. Bars represent mean profile abundance of each PA in all identified subunits of CL molecules. **d** The double-bond distribution in CLs. Bars represent the mean profile abundance of total double-bond counts in all identified CL species. **e** Relative abundance of CL species normalized to internal standard CL(14:0/14:0/14:0/14:0) (*n* = 5). **f** CL in BLF of LCMV-clone13-infected mice at 7 dpi assessed by fluorometric assay (*n* = 6). **g–k** MDCK cells were infected, treated with LPS (10 μg/mL) or hypoxic environment (1% O_2_/5% CO_2_) for 24 h. **g** Content of CL (*n* = 5). **h** Transmission electron microscopy (TEM) examination. Mitochondria (arrowheads), vacuole formation (double arrows), filamentous particle formation and viral particles (arrows). **i** ATP in supernatant assessed using Cell-Glo ATP assay (*n* = 6). **j, k** MitoTracker Green and MitoSOX red mitochondrial superoxide indicator (**j**) or JC-1 dye (**k**) were used for assessing production of superoxide by mitochondria (**j**) or mitochondrial depolarization (**k**) (*n* = 5). **l** Mice at 1 dpi were i.p. injected with CL (100 μg/kg) and analysed at 5 dpi (*n* = 3). **m** Mice were i.v. given 500 μg of anti-CD1d antibody at 1 dpi and analysed at 4 dpi (*n* = 3, 5). **n** Schema of antibiotics treatment in **o** and **p** (*n* = 10). **o** Bacteria load in stool and BLF. **p** Frequency of IL-17A^+^ γδ T (left) and number of γδ T cells (right) in lungs. Data are combined from two or three independent experiments and represented as mean ± SEM. *P* values were determined using two-tailed unpaired Student’s *t*-test (**e, f, l, m, o, p**) or one-way ANOVA (**g, i, j, k**). Source data are included in Source Data file.

Host-derived molecules released by cells under stress from virus-induced lung tissue damage may contribute to γδ T cell activation. Since CLs were previously identified as CD1d-presenting lipid antigen^19^, we next profiled and structurally characterized CLs fractions extracted from BLF of mock- and pdmH1N1-infected mice at 5 dpi by reverse phase liquid chromatography-mass spectrometry (LC-MS). Parallel reaction monitoring (PRM) was applied for quantitation of CL molecules due to the presence of isomeric molecules. Eight different CL lipid molecules were detected with CL(36:4/36:4) being dominantly represented (Fig. 5a–c and Supplementary Fig. 5). CLs are a class of dimeric phospholipids that support mitochondrial cristae formation and ATP synthase function, and can be released by stressed cells acting as a “danger signal” during autoimmunity or infections^40,41^. The influenza-infected BLF profile resembled that typically found in murine mitochondria^42^, suggesting the release of CLs possibly from mitochondria of infected lung cells. Moreover, the double-bond distribution in CLs of BLFs from influenza-infected mice showed a certain degree of mild shifting towards less unsaturated molecules compared with those from mock-infected controls (Fig. 5d). CL species such as CL(36:4/36:4) are significantly increased in BLF of infected mice when compared to mock controls (Fig. 5e). We also assessed total CL levels in the BLFs of mice infected intranasally (i.n.) with lymphocytic choriomeningitis virus (LCMV) and found that, compared with those of mock-infected controls, BLFs of LCMV-infected mice had profoundly increased CL (Fig. 5f), suggesting that increased CLs could be a common characteristic of lung viral infections.

Consistently, CL content detected in the culture medium of influenza-infected Madin-Darby Canine Kidney (MDCK) cells, an epithelia-derived cell line, which can be efficiently infected by influenza virus, was significantly higher than that of mock-infected or LPS-stimulated cells. Subjecting cells to hypoxic conditions (1% O_2_) also induced release of CL to the culture medium (Fig. 5g). In accordance with CL being derived from damaged mitochondria, ultrastructural alterations of mitochondria were observed in influenza-infected cells as shown by transmission electron microscopy (TEM) analysis (Fig. 5h). In assessing alterations of core mitochondrial metabolic functions, we found that influenza infection of MDCK cells culminated in a 40% decrease in extracellular ATP levels (Fig. 5i), consistent with increased mitochondrial superoxide and membrane potential loss as reflected by signals from MitoSOX Red and JC-1 (Fig. 5j, k). These results indicate that influenza infection induces metabolic stress and hypoxia, resulting in release of endogenous lipids from host cells in the lung.

Since mouse γδ T cells were previously shown to respond to CLs presented by CD1d expressed on antigen-presenting cell (APCs)^19,21^, we next determined whether CD1d-associated lipid antigens such as CLs can induce γδ T cells to produce IL-17A in vivo by injecting mixed forms of CL species intraperitoneally (i.p.) into pdmH1N1-infected mice. Administration of CLs significantly increased IL-17A production by lung γδ T cells when compared with injection of vehicle control (Fig. 5l). Moreover, treatment of anti-CD1d blocking mAb markedly inhibited IL-17A production by γδ T cells in infected mice (Fig. 5m). As both influenza virus and LCMV do not carry lipids, we next examine if microbiota-derived lipids play a role in activating γδ T cells for IL-17A production. Mice were treated by antibiotics via both intranasal and oral routes (Fig. 5n). However, although antibiotics remarkably reduced bacterial load in stool and airways (Fig. 5o), IL-17A production and number of γδ T cells were not affected (Fig. 5p). Taken together, our results indicate the involvement of endogenous lipids in mediating γδ T cell activation and IL-17A production via CD1d-dependent presentation.

### γδ T cell IL-17A production requires CD1d-dependent antigen presentation

To identify the key cellular components acting as lipid antigen-presenting cells for γδ T cell activation, we examined CD1d expression on different immune cell types in the lung tissue of pdmH1N1-infected mice and found most highly expressed CD1d on B-1a cells among conventional B cells, CD4^+^ T cells, CD8^+^ T cells, NK cells, dendritic cells (DCs), neutrophils, and macrophages (Supplementary Fig. 6a–c). Among B cell subsets, lung B-1a cells and splenic marginal zone (MZ) B cells were found to express the highest levels of CD1d (Supplementary Fig. 6d, e). However, MZ B cells were present only in the spleens but not lungs, MedLNs or peritoneal cavities of infected mice (Supplementary Fig. 6f). When CFSE-labelled cavity B-1a cells were i.v. transferred into infected mice at 1 dpi, we detected upregulated CD1d expression on CFSE^+^ B-1a cells following their migration into lung tissues (Supplementary Fig. 6g, h).

**Fig. 6:**
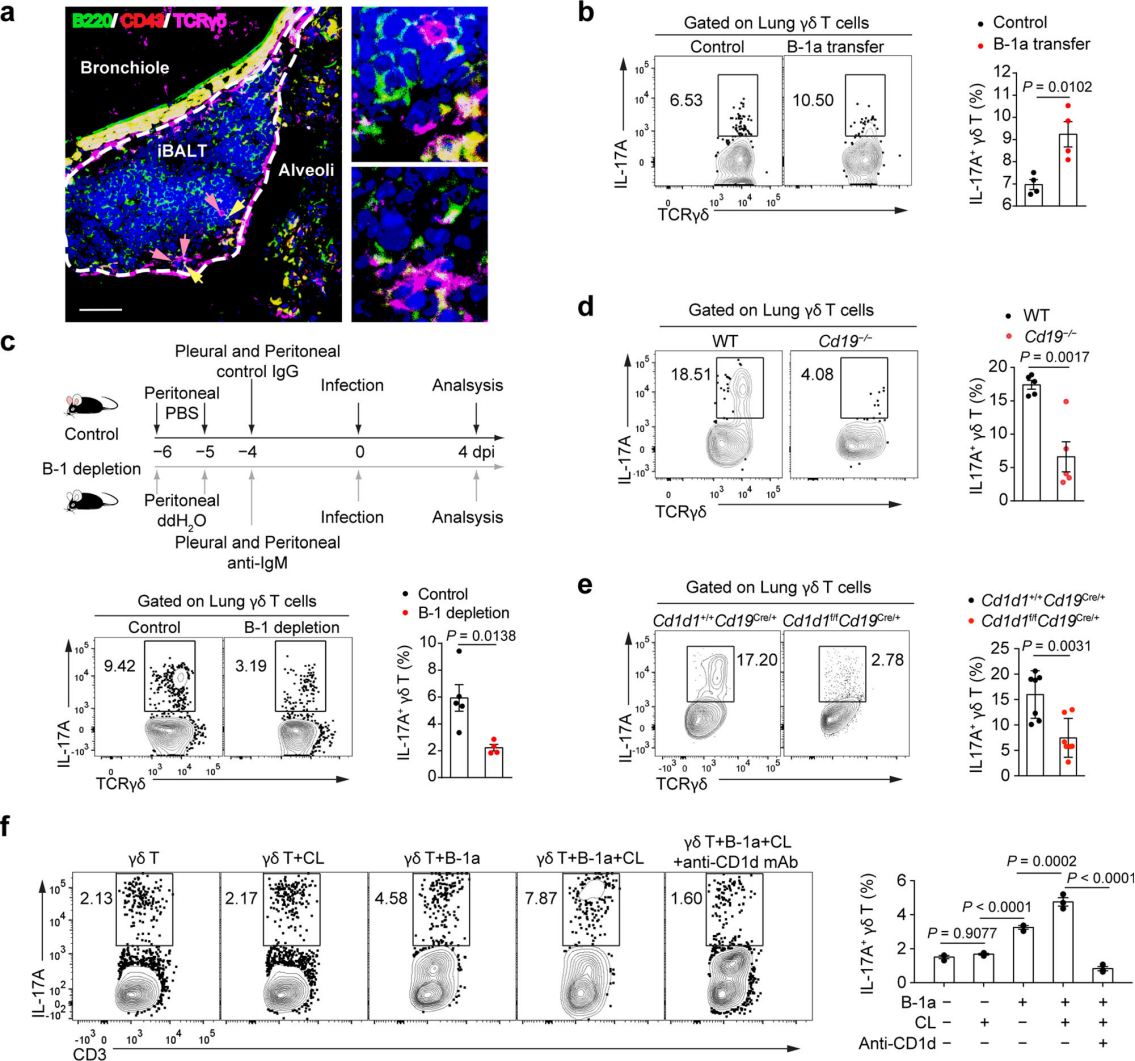
γδ T IL-17A production requires CD1d-dependent antigen presentation. **a** Confocal immunofluorescence microscopy of lungs from pdmH1N1-infected mice at 3 dpi, stained for B220 (green), CD43 (red), TCRγδ (purple), and DAPI (blue). Outlined areas (left row) are enlarged 10× at right. Scale bar, 50 μm. **b** Mice at 1 dpi were i.v. injected with 5 × 10^5^ CL-preloaded B-1a cells and analysed by flow cytometry at 5 dpi. Flow cytometry plots (left) and cumulative data (right) showing frequency of IL-17A^+^ cells in gated lung CD3^+^TCRγδ^+^ cells (*n* = 4). **c** Schema of B-1a cell depletion. Mice were infected with pdmH1N1 and analysed at 4 dpi. Representative flow cytometry plots (left) and cumulative data (right) showing frequencies of IL-17A^+^ cells in gated lung CD3^+^ TCRγδ^+^ cells (*n* = 5, 4). **d** CD19-deficient (*Cd19*^−/−^) and WT mice were infected with pdmH1N1 and analysed at 5 dpi. Representative flow cytometry plots (left) and cumulative data (right) showing frequencies of IL-17A^+^ cells in gated lung CD3^+^TCRγδ^+^ cells (*n* = 5). **e** CD*1d1*^+/+^CD*19*^Cre/+^ and control CD*1d1*^f/f^CD*19*^Cre/+^ mice were infected with pdmH1N1 and analysed at 5 dpi. Representative flow cytometry plots (left) and cumulative data (right) showing frequencies of IL-17A^+^ cells in gated lung CD3^+^TCRγδ^+^ cells (*n* = 7). **f** Purified γδ T cells (1 × 10^5^/well) were cultured 1:4 with B-1a cells from naïve C57BL/6 mice with or without CL, and with 10 μg/mL anti-CD1d blocking antibody or isotype control antibody for 3 days. IL-17A production in γδ T cells was determined by flow cytometry. Representative flow cytometry plots (left) and cumulative data (right) showing frequency of IL-17A^+^ cells in gated γδ T cells (*n* = 3). Data are combined from two or three independent experiments and are represented as mean ± SEM. *P-*values were determined using two-tailed unpaired Student’s *t*-test (**b**–**e**) or one-way ANOVA (**f**). Source data are included in Source Data file.

Since B-1a cells were proximally close to γδ T cells in lung tissue of influenza-infected mice as evidenced by immunofluorescence microscopy (Fig. 6a), we investigated a possible role for B-1a cells in activating Tγδ17 cells in vivo. B-1a cells were preloaded with CL and i.v. transferred into infected C57BL/6 mice at 1 dpi. Four days post-transfer, lung γδ T cells in mice receiving CL-loaded B-1a cells produced significantly more IL-17A than those in control mice receiving no B-1a cells (Fig. 6b). To further verify the critical involvement of B-1a cells in γδ T cell activation, we depleted B-1a cells via i.p. injection of monoclonal anti-IgM antibodies (Supplementary Fig. 6i). Consistently, B-1a depletion resulted in significantly reduced IL-17A production in γδ T cells when compared with control mice upon viral infection (Fig. 6c). Furthermore, we analysed CD19-deficient (*Cd19*^−/−^) mice that lack B-1a cells^43,44^. Following influenza infection, significantly reduced IL-17A production was detected in lung γδ T cells from *Cd19*^−/−^ mice when compared to wild-type controls (Fig. 6d). The IL-17A production in γδ T cells was also significantly reduced in *Cd1d1*^f/f^*Cd19*^Cre/+^ mice compared with control animals, indicating that other immune cells cannot compensate for the absence of CD1d expression on B cells (Fig. 6e). In vitro, B-1a cells preloaded with CL promoted substantially more IL-17A production by co-cultured γδ T cells than CL or B-1a cells alone, and this was abrogated by the addition of anti-CD1d blocking mAb (Fig. 6f). Together, these results strongly suggest that B-1a cells can directly activate γδ T cells to produce IL-17A via CD1d-mediated self-lipid antigen presentation in vivo.

Among B cell subsets involved in early antibody production in the lungs of infected mice, we identified CD19^+^CD43^+^CD5^+^ B-1a cells as the predominant B cell subset present in lung infiltrates and producing IgM specific for influenza surface protein HA at 4 dpi when IgM was the major isotype, in contrast to HA^+^IgG1^+^ B-1a cells that were barely detected. Moreover, lung HA^+^IgM^+^ B-1a cells displayed a CD38^+^CD138^+/−^GL-7^−^ plasmablast phenotype (Supplementary Fig. 7a). When γδ T cells were silenced via injection of anti-γδTCR mAb 3 days (Supplementary Fig. 7b, c) or thymectomy was performed 24 h, before viral infection, differentiation of B-1a cells was severely impaired in mice as revealed by marked decline in frequencies of CD19^+^CD43^+^CD5^+^IgM^+^HA^+^ (Supplementary Fig. 7d) and CD38^+^CD138^+^HA^+^ (Supplementary Fig. 7e) B-1a cell populations in the infected lungs. Consistently, we found that infection-induced total IgM, flu-specific, and phosphorylcholine (PC)-specific IgM titers were substantially reduced in the airways of anti-γδTCR-treated or thymectomized mice (Supplementary Fig. 7f), suggesting an overall impairment of natural antibody response in these mice. In addition, we asked if γδ T-cell-derived IL-17A is important for the observed plasmacytic differentiation of B-1a cells. We found that following infection, *Il17a*^−/−^ mice, compared with wild-type mice, had significantly decreased frequencies of lung HA^+^ B-1a cells (Supplementary Fig. 7g) and HA^+^CD138^+^ B-1a (Supplementary Fig. 7h). Transfer of wild-type but not *Il17a*^−/−^ γδ T cells into infected *Il17a*^−/−^ mice restored the number of lung HA^+^ B-1a cells (Supplementary Fig. 7g) and their CD138 expression (Supplementary Fig. 7h). Analysis of single-cell suspensions from infected lung tissue by ELISPOT assay confirmed significantly increased influenza virus-specific and total IgM-secreting cells in mice that received wild-type but not *Il17a*^−/−^ γδ T cells (Supplementary Fig. 7i). Serum titers of virus-specific and total IgM were also greater in *Il17a*^−/−^ mice that received wild-type but not *Il17a*^−/−^ γδ T cells or mice that received no cell transfer (Supplementary Fig. 7j). Together, these data identify an important role of Tγδ17 cells in promoting plasmacytic differentiation of B-1a cells and their natural antibody production in response to lung viral infection.

### Human lung γδ T cells correlate with pneumonia severity

**Fig. 7:**
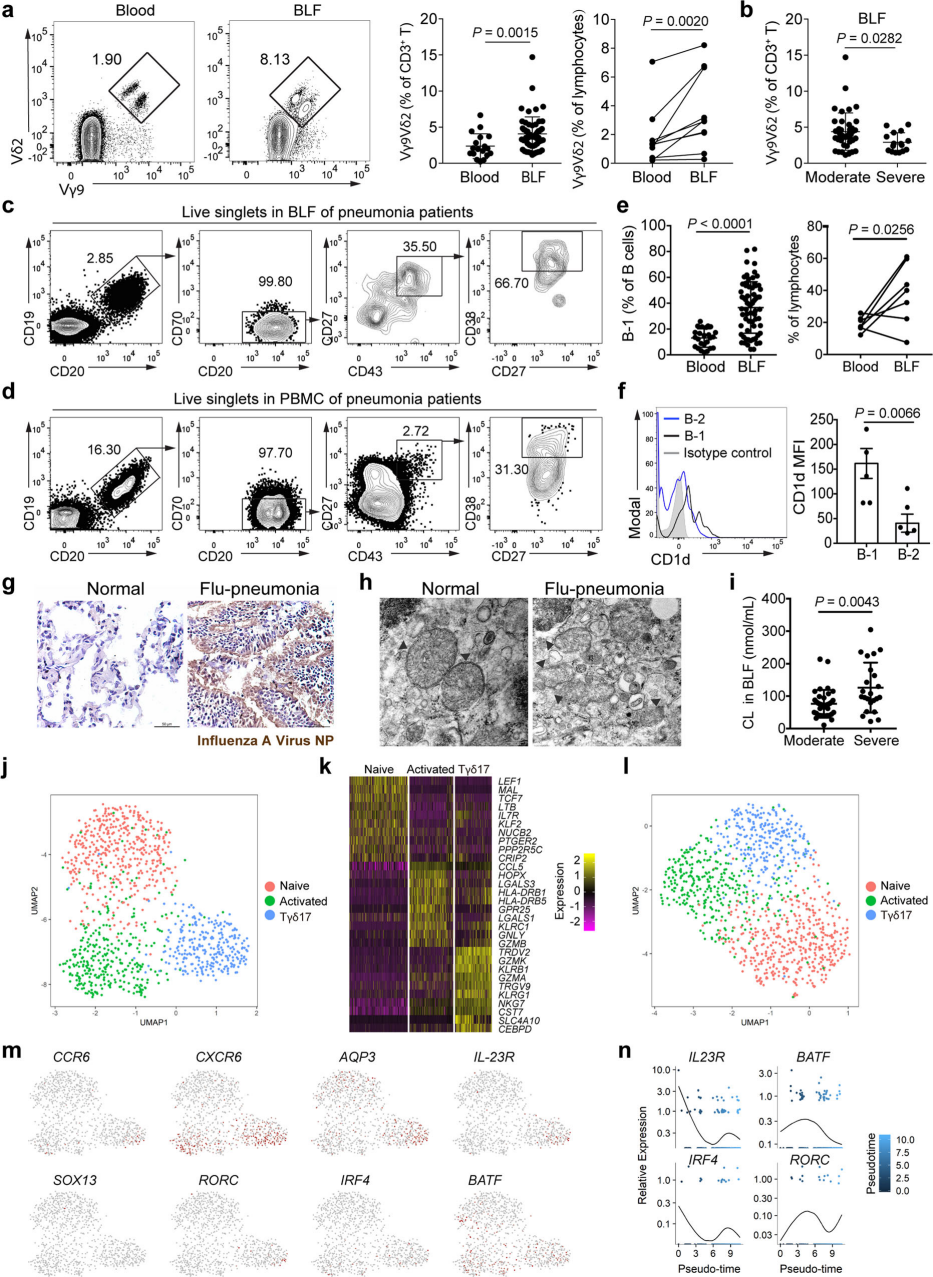
Correlations between human lung γδ T cells and pneumonia severity. **a** Representative plots (left) and cumulative data (right) showing prevalence of Vγ9Vδ2 cells in Blood (*n* = 18) and BLF (*n* = 57, paired samples *n* = 10) of pneumonia patients. Left: *P*-values calculated by Mann–Whitney U test; Right: *p*-values calculated by Wilcoxon matched-pairs signed rank test. Data are represented as mean ± SD. **b** Prevalence of Vγ9Vδ2 cells in patients diagnosed as moderate (*n* = 41) and severe (*n* = 16) pneumonia. *P*-values calculated by Mann–Whitney U test. Data are represented as mean ± SD. **c, d** Gating strategy of B-1 cells in BLF (**c**) and PBMC (**d**) of pneumonia patients. **e** Prevalence of B-1 cells in Blood (*n* = 25) and BLF (*n* = 66, paired samples *n* = 7) of pneumonia patients. Left: *P*-values calculated by Mann–Whitney U test; right: *P*-values calculated by Wilcoxon matched-pairs signed rank test. Data are represented as mean ± SD. **f** CD1d expression on B-1 and B-2 cells in BLF of pneumonia patients (*n* = 5). Two-tailed unpaired Student’s *t-*test. **g** Immunohistochemical staining of Influenza A Virus nucleoprotein (NP) in the lung tissues from influenza-infetced pneumonia patient. Scale bar, 50 μm. **h** Lung tissues of influenza-infected pneumonia patient were fixed and subjected to TEM examination. Mitochondria (arrowheads) were identified in the images. Scale bar, 200 nm. **i** Content of CL in BLF of patients diagnosed as moderate (*n* = 37) and severe (*n* = 23) pneumonia was assessed by fluorometric assay. *P*-values calculated by Mann–Whitney U test. Data are represented as mean ± SD. **j** UMAP clustering of human γδ T cells present in the BLF of pneumonia patients. **k** Heatmap of highly variable genes in each cluster. **l** UMAP clustering using genes found to be variably expressed in mouse γδ T cells demonstrates that these three clusters observed include a significant degree of conservation between mouse model and human disease. **m** UMAP visualization of the expression profiles for key genes. **n** Pseudotime trajectory analysis of genes in Tγδ17 cluster demonstrates that *IRF4* and *IL23R* expression are tightly correlated, while *RORC* and *BATF* are not appreciably linked. Source data are included in Source Data file.

To determine whether the protective role for γδ Τ cells during infection in mice might extend to humans, we analysed γδ T cells from PBMCs and BLF of patients with community-acquired pneumonia (CAP), and examined their frequency and relationship with CAP severity. Vγ9Vδ2 cells, a major IL-17A-producing γδ T cell population in human (Fig. 4i), significantly increased in BLF, associated negatively with pneumonia severity (Fig. 7a,b). We compared the phenotype of blood and BLF B-1 cells from pneumonia patients and detected B-1 cells from both sites exhibiting a phenotype of CD19^+^CD20^+^CD27^+^CD43^+^CD70^−^ (Fig. 7c, d)^45^. Compared to PBMCs, the frequency of B-1 cells in BLF also significantly increased (Fig. 7e). In BLF, B-1 cells expressed increased levels of CD1d compared with B-2 cells (Fig. 7f). Moreover, we observed ultrastructural alterations of mitochondria in influenza-infected lungs from patients as shown by TEM analysis (Fig. 7g, h). Consistently, CL content detected in the BLF from severe CAP was significantly higher than that from moderate CAP (Fig. 7i), suggesting a correlation between tissue damage and CL release. We further performed scRNA-seq with pooled T cells from 5 CAP patients. UMAP clustering (Fig. 7j) and heatmap of the highly variable genes (Fig. 7k) of human γδ T cells present in the BLF of pneumonia patients revealed the presence of three main populations, including naïve/not yet activated cells, activated cells with cytotoxic potential, and Tγδ17 cells that were highly enriched for usage of Vγ9 and Vδ2. UMAP clustering using the genes found to be variably expressed in mouse γδ T cells illustrated that key drivers behind the 3 observed clusters included a significant degree of conservation between mouse and human (Fig. 7l). A small portion of these cells in population two are *AQP3*^*+*^*IL23R*^+^ and *RORC*^+^, suggestive of Tγδ17 identity (Fig. 7m). Pseudotime trajectory analysis of cluster Tγδ17 further showed that *IRF4* and *IL23R* expression were tightly correlated, while *RORC* and *BATF* were not appreciably linked (Fig. 7n and Supplementary Fig. 8). Therefore, these correlations may possibly indicate a similar mechanism for Tγδ17 cell activation mediated by B-1 cells in lungs of pneumonia patients.

## Discussion

Recent studies have highlighted a critical role of γδ T cells in protective host defense^5–8,10^, but the cellular and molecular mechanisms underlying activation and subsequent differentiation of γδ T cells in the lungs remain poorly understood. Here we reveal that thymus-derived γδ T cells arise in the lung from day 1 following pdmH1N1 influenza infection and act as the predominant producers of local IL-17A. IL-17A production by lung γδ T cells is induced by endogenous lipids presented by B-1a cells and regulated by a T cell-intrinsic TCR-IRF4 axis. Reciprocally, lung Tγδ17 cells promote plasmacytic differentiation of B-1a cells and enhance their natural antibody production. Together, these findings define a role of host-derived lipids that function as danger signals in the setting of influenza virus infection and orchestrate the functional interaction between lung γδ T cells and B-1a cells in a feed forward loop to provide an early innate protection.

Studies have shown that levels of lipid constituents are increased in respiratory tract and play a proinflammatory role in lung epithelial cells during influenza infection^2,3^. Here, we reveal that endogenous lipids can be released into the extracellular milieu during influenza infection and subsequently activate γδ T cells for rapid IL-17A induction. Our findings are particularly important in light of recent evidence showing that during influenza viral infection, lipid components from unknown source are involved in NKT-cell activation in MedLN^22^. Previous studies demonstrated that influenza virus PB1-F2 induces apoptosis, increased mitochondrial permeability and content release^46,47^. CLs, derived from stressed cells or microbiota, could potentially be presented by APCs during infection and inflammation^19,21,40,41^. Here we show that influenza infection directly induces the release of endogenous CLs from both host cells in vivo and epithelial cells in vitro, which is consistent with ruptured structure of mitochondria in pdmH1N1-infected cells by TEM analysis. Moreover, LCMV infection also induces the release of CL in BLF, suggesting the possibility of a general mechanism across pulmonary viral infections. Antibiotics treatment does not affect lipids-mediated IL-17A production in γδ T cells, further supporting the endogenous nature of the lipids. Compared with the CL species identified in BLF of mock-infected controls, CLs in BLF of infected mice show mild shifting towards less unsaturated status regarding carbon double bonds distribution in their structure, hence suggesting a high degree of saturation in that of infected mice. Moreover, hypoxic condition results in increased CL release from MDCK cells, indicating a possible role of hypoxia in inducing CL release during lung infections. It is reported that the types of lipid antigens recognized by TCRs of γδ T or NKT cells are profoundly different^18^. Indeed, the identification of endogenous lipids that initiate Tγδ17 cell response in lung tissues at very early stage of influenza infection may provide a possible mechanistic explanation for the activation of NKT cells at a later stage in MedLN^22^. In addition to CLs, other lipid components may be involved in the activation of γδ T cells in lung under the influenza infection condition. Thus, further elucidation on the exact mechanism that mediates lipids release and the glycolipid antigen repertoire recognized by γδ T cells should provide new insight in understanding the immune responses induced by live attenuated vaccines delivered via the mucosal route.

To determine molecular and phenotypic features of lung γδ T cells, our scRNA-seq analysis shows that lung Tγδ17 cells exhibit a unique transcriptome suggestive of functional maturity associated with TCR activation and thymic origin. The high levels of TCRγδ and CD3 expression on Tγδ17 cells prompted us to investigate whether TCR signaling plays a critical role in determining differentiation of γδ T cells. Unlike MHC molecules presenting peptide antigens, CD1 family molecules present lipids and microbial metabolites, thereby providing a broad survey of self and foreign antigens encountered by the host^48^. Recent advances have been made in the identification of natural lipid antigens derived from either host (endogenous) or foreign (exogenous) pathogens^18^. Thus, the γδ T cell receptors, when presented with the lipid ligands in the context of CD1d, more closely resemble pattern recognition receptors, rather than the adaptive αβ T cell receptors with high diversity and specificity.

The transcription factor IRF4 is required for both development and function of various immune cells, including plasma cells, regulatory T cells and several helper T cell subsets^38,49,50^. IRF4 expression is strictly controlled by TCR affinity and acts to regulate T cell functions in a dose-dependent manner^51,52^. IRF4 can directly bind to the *Il17a* promotor and induce *Il17a* and *Rorγt* gene expression in CD4^+^ T cells^52^. However, it has been unclear whether and how the strength of TCR signaling regulates the abundance of IRF4 expression and IL-17A production. Here we show that *Irf4*^−/−^ mice fail to mount a productive pulmonary Tγδ17 cell response during influenza challenge. Moreover, we demonstrate that IRF4 expression is tightly controlled by TCR signaling strength in γδ T cells and IRF4 promotes IL-17A production in a manner dependent on TCR strength. These results demonstrate that the TCR-IRF4 axis critically regulates IL-17A production. Although other mechanisms may be involved in mediating IL-17A production in γδ T cells, this study has identified γδTCR-IRF4 axis as the crucial mechanism of Tγδ17 induction in the context of influenza infection. Here, we have also observed considerable variations of Tγδ17 cell frequencies in infected lung tissues compared with other lymphoid organs, which may reflect the functional heterogeneity of different γδ T cell subsets with distinctive cytokine production or usage of specific Vγ chain segments, a notion further supported by our current findings that lung γδ T cells have much higher frequencies of Vγ4, Vγ5 and Vγ6 subsets enriched in Tγδ17 cells.

Analysis at the single-cell level confirmed a functional heterogeneity and demonstrated that the expression of CXCR6, a receptor involved in relocating tissue-resident T cells to the airways and assists in their retaining in the tissue^53^, identifies Tγδ17 cells. Interestingly, we also identified a strong link between AQP3 and IL-17A in γδ T cells, which has been independently associated with tissue origins. AQP3 is one of thirteen members of the aquaglyceroporin family that transports water and other small molecules, including hydrogen peroxide (H_2_O_2_) and glycogen, across the cell membrane^54^. It has been shown that AQP3-mediated H_2_O_2_ uptake regulates trafficking of CD4^+^ T cells in cutaneous immune reactions^55^. AQP3 has also been reported to mediate H_2_O_2_-dependent expression of IL-6 and TNF in colonic epithelial cells in response to environmental stress, a condition linked to epithelial wound repair, defense against infections, and inflammation^56^. Notably, *Aqp3* is among the top ranking genes that are induced following the in vitro exposure of naïve T cells to Th17 polarizing conditions including transforming growth factor β1 and IL-6^57^. Thus, further investigation is warranted to determine whether AQP3 mediates the adaptation of γδ T cells to hypoxic conditions in influenza-infected lungs.

Many studies have reported the presentation of lipid antigens by CD1d-expressing APCs, including DCs, macrophages, neutrophils and B cells^19–21,23,58–60^. B cells are known to use multiple pathways to capture lipid antigens. Previous studies have shown that BCR-mediated uptake of the prototypical NKT-cell antigen, α-GalCer, elicits cognate NKT help for lipid-specific B cells in vivo and in vitro^24,61^. However, B cells also capture, internalize and present lipid antigens to NKT cells via apolipoprotein E (apoE), and the low-density lipoprotein receptor (LDL-R), which initiates innate help by NKT cells to stimulate polyclonal B cell activation^60^. In this study, we show that presentation of lipid antigen by lung B-1a cells to γδ T cells induces concomitant differentiation of both virus-specific and PC (a hapten present on both mammalian cells and many bacteria)-specific B-1a cells into CD138-expressing plasmablasts^62,63^. Thus, consistent with previous findings that B-1a response in MedLN lacked antigen specificity in response to influenza infection^64^, it is plausible to reason that B-1a-γδ T interaction preferentially favors innate and noncognate types of immune responses. As a result, B-1a and γδ T cells cooperate to generate an innate humoral response preceding a more specific and evolved adaptive response. Nevertheless, future studies are needed to determine whether lung Tγδ17 cells exert any adaptive property in terms of their homeostatic maintenance and secondary response to the same stimulus.

It has been proposed that layers of progressively more advanced populations of lymphocytes create an evolutionarily “layered immune system”, in which B-1a and γδ T cells appear to occupy equivalent positions with their adaptive counterparts in primitive layer and are expected to respond rapidly to invading pathogens^65^. Here, we show that innate B-1a and Tγδ17 cells interact in a fashion reminiscent of adaptive B-2 and Th17 cells. Recent studies have provided supportive evidence for such B-1a-γδ T interaction. First, the antigen receptor repertoires of B-1a and γδ T cells are conserved yet overlapping to recognize similar ligands such as certain phospholipids, and this allows the two cell types to influence the immune responses of each other^66–68^. Second, B-1a cells express the MHC I-like molecule CD1d, which can be recognized by γδTCR^19,21^. Third, both B-1a and γδ T cells, unlike their adaptive counterparts, acquire a semi-activated state under homeostatic conditions and produce respectively natural antibodies and cytokines, but can undergo further differentiation during infection and inflammation^5,6,69^, which enable them to provide immediate help to each other under both naïve and inflammatory conditions. However, to what extent this innate B–T interaction requires cognate recognition and whether co-stimulatory signals such as CD40L-CD40 and B-7-CD28 are involved remain to be investigated. Addressing these key questions may facilitate the development of new strategies to modulate innate B–T interaction for therapeutic benefits in infection, autoimmunity, and vaccine development.

## Methods

### Mice

Female mice between 6 and 8 weeks of age on C57BL/6 background were used. IL-17A-deficient (*Il17a*^−/−^) mice were obtained from Dr. Yoichiro Iwakura at The Institute of Medical Science, The University of Tokyo, Japan. IRF4-deficient (*Irf4*^−/−^) mice were provided by Dr. Tak Wah Mak at University Health Network, Toronto. C57BL/6 wild-type mice, B6.SJL-PtrcaPep3b/BoyJ (CD45.1) mice, C.Cg-*Cd19*^tm1(cre)Cgn^*Igh*^b^/J (*Cd19*^−/−^), *Cd1d1*^f/f^, and *Cd19*^Cre/+^ mice were purchased from The Jackson Laboratory (Bar Harbor, ME, USA). All the mice were housed and bred under specific pathogen-free conditions at the animal facilities of The University of Hong Kong, Shenzhen University School of Medicine, or Bioprocessing Technology Institute, Agency for Science, Technology and Research (A*STAR), Singapore. Mice were housed under specific pathogen-free conditions with a 12 h light/dark cycle, at a temperature of 22 ± 2 °C, and a relative humidity of 50 ± 5%. Mice were fed a standard mouse chow diet.

### Virus preparation and infection

Influenza type A virus, mouse-adopted mutant strain of pandemic H1N1 (HK/415742/09-Mut), was provided by Dr. Bo-Jian Zheng at Department of Microbiology, The University of Hong Kong, and propagated in the allantoic cavity of 10-day-old embryonated hens’ eggs at 37 °C with 65% humidity for 48 h. Allantoic fluid was collected and stored in aliquots. For influenza virus challenge experiments, mice were housed in biosafety level-2 individual ventilation cages and given free access to food and water. The 50% lethal dose (LD_50_) of pdmH1N1 was determined in C57BL/6 mice after serial dilution of the viral stock from embryonated hens’ eggs, and LD_50_ of pdmH1N1 was adopted in viral challenge experiments. After anesthetized with isoflurane, mice were i.n. challenged with 30 μL of virus diluted in phosphate-buffered saline (PBS). Weight loss and survival were monitored for 14 successive days. Mice were sacrificed at the indicated time points for examination. For LCMV infection, mice were infected with 1 × 10^6^ PFU and 5 × 10^5^ PFU of LCMV-clone13 via the i.v. and i.n. routes, respectively. All animal experiments were approved by Institutional Committee on the Use of Live Animals in Teaching and Research of The University of Hong Kong, Animal Ethical and Welfare Committee of Medical College at Shenzhen University, or A*STAR Biological Resource Centre Institutional Animal Care and Use Committee.

To prepare inactivated virus, the allantoic fluid was concentrated and purified in a 10–50% sucrose gradient by centrifugation at 25,000 × *g*, 4 °C for 2 h. The purified virus was inactivated with 0.25% formalin (v/v) at 4 °C for 7 days. Further purification was performed with Amicon Ultra Membrane (4208) (Millipore, Billerica, MA, USA), with a molecular weight cutoff at 30 kDa. The products were resuspended in PBS. Inactivation of the virus was confirmed by the absence of cytopathic effects and detectable HA in the supernatant of two consecutive 50% tissue culture infectious dose (TCID_50_) assays.

### Human samples

The participants of the study were a part of the patients’ cohort we have previously reported^70^. Information on selected patients is described in Supplementary Table 1. The study was approved by the Medical Ethics Committee of Guangzhou Women and Children’s Medical Centre. Flexible fiberoptic bronchoscopy operations were performed according to the International Ethical Guidelines for Research Involving Human Subjects as stated in the Helsinki Declaration. The legal guardians of all participants provided informed written consents. Healthy PBMCs were isolated from buffy coat blood from healthy donors collected by the Hong Kong Red Cross with written consent. All experiments using PBMCs from healthy donors were approved by Institutional Review Board of The University of Hong Kong/ Hospital Authority Hong Kong West Cluster Institutional Review Board (HKU/ HA HKW IRB).

### Mouse injection

Mice were given i.p. injection of 200 μg of anti-CD1d (1B1, Biolegend) blocking antibody diluted in 200 μL of PBS, and intrapleural injections of 150 μg of anti-CD1d blocking antibody diluted in 60 μL of PBS to each side at 1 dpi. Mice that received injections of isotype-matched antibody served as controls. Mice were sacrificed and lung tissues were collected at 4 dpi. Mice were i.p. injected 200 μg of anti-TCRγδ (GL3, Biolegend) or isotype control antibodies 3 days before pdmH1N1 infection. B-1a cell depletion was achieved using protocols previously reported with some modifications^71,72^. Briefly, 6-week-old mice were treated with i.p. injections of 3 mL water daily for two days, and control mice received the same volume of PBS. The next day of the second water injection, mice were given i.p. injection of 200 μg of purified anti-mouse IgM (RMM-1, Biolegend) antibody diluted in 200 μL of PBS, and intrapleural injections of 150 μg of purified anti-mouse IgM (RMM-1) diluted in 60 μL of PBS to each side. Mice that received injections of isotype-matched antibody served as controls. Mice were allowed for 4 days to rest for influenza infection and another 4 days for flow cytometric analysis. The efficiency and specificity of B-1a depletion in various organs were examined at 4 dpi.

### Bone marrow chimeras

Recipient B6.SJL-PtrcaPep3b/BoyJ (CD45.1) mice were given 700 cGy whole-body irradiation and allowed to rest for 7 h, followed by bone marrow reconstitution. Recipient mice were reconstituted with 5 × 10^6^ bone marrow cells including a 1:1 ratio of wild-type CD45.1^+^ and *Irf4*^−/−^ CD45.2^+^ bone marrow cells. This reconstitution mixture resulted in mice in which all the CD45.2^+^ cells were deficient in IRF4 and all the CD45.1^+^ cells were IRF4 sufficient. Graft reconstitution was evaluated by peripheral blood analysis of congenic markers expressed by lymphocytes. Recipient mice were allowed 8–10 weeks for reconstitution before infection with influenza virus.

### Quantitative RT-PCR

γδ T cells were subjected to RLT lysis buffer followed by RNA extraction using the RNeasy kit from Qiagen according to the manufacturer’s instructions. RNA concentrations were measured using Thermo Scientific NanoDrop™ spectrophotometers. RNA was reverse-transcribed to cDNA using the PrimeScript^TM^ RT reagent kit (TaKaRa). Quantitative RT-PCR was conducted with the Applied Biosystems 7900HT Fast Real-Time PCR System, using SYBR^®^ Premix Ex Taq^TM^ (Perfect Real Time) (TaKaRa). The expression of mRNA encoding detected genes was normalized relative to that of the mRNA encoding the internal standard hypoxanthine-guanine phosphoribosyltransferase (*Hprt*). All experiments were related to controls via the ΔΔ C_T_ method.

### Flow cytometry and antibodies

Single-cell suspensions of mouse spleen, lymph node, thymus and lung were treated with red blood cell lysis buffer, then blocked with purified anti-mouse CD16/32 (clone 93) to reduce nonspecific labeling, and the cells were thereafter stained with Live/Dead Zombie Aqua™ Fixable Viability dye (Biolegend) to stain for dead cells. Total lung lymphocytes were enriched by Percoll density centrifugation and collected from the interface of the 40/70% Percoll gradient. Anti-TCR Vγ7 (GL1.7) was a gift from Dr. Rebecca L. O’Brien (National Jewish Health, Denver, CO) and mAb 17D1 was a gift from Dr. Robert E Tigelaar and Dr. Julia M. Lewis (Yale School of Medicine, Connecticut, USA). FITC-conjugated goat antibody to rat (anti-rat) IgM (MRM-47) for detecting mAb 17D1, and normal hamster IgG for blocking were purchased from BioLegend. In the absence of other antibodies to TCR, mAb 17D1 binds to an epitope formed by the combination of Vγ5 and Vδ1 but does not bind either of these V regions individually. However, if anti-Vδ (GL3) binds the TCR first, an epitope on Vγ6Vδ1 TCRs that can bind mAb 17D1 is exposed^73^. Antibody dilution of 1:100–1:400 was used for flow cytometry analysis. Intracellular staining and intranuclear staining were performed using the BD Cytofix/Cytoperm™ Fixation/Permeabilization Kit and FoxP3 staining kit (eBioscience) according to the manufacturer’s instructions, respectively. For intracellular cytokine staining, cells were stimulated with PMA (200 ng/mL, Sigma), Ionomycin (500 ng/mL, Sigma), and monensin (1:1000 dilution, Biolegend) at 37 °C for 4 h, followed by fixation with the Fixation/Permeabilization buffer solution. Antibody information is summarized in Supplementary Table 2. Stained cells were acquired on a BD FACSAria SORP (Becton Dickinson) and analysed using FlowJo software (TreeStar). Debris and dead cells with lower FSC and SSC were excluded. FSC-H versus FSC-A was used to identify the singlet events. The Live/Dead Zombie Aqua™ Fixable Viability dye (Biolegend) negative live cells were used for further subgating (Figure exemplifies the gating strategy is provided in the Supplementary Fig. 6a).

### CHIP assay

Freshly purified γδ T cells stimulated with plate-bound antibody to the co-receptor CD28 (1 μg/mL anti-CD28) and various concentrations of anti-CD3ε were prepared for CHIP assay using ChIP Assay Kit (Beyotime) according to manufacturer’s instructions. Cell lysates were sonicated, followed by precipitation with anti-IRF4 or control rabbit IgG. Quantification of IRF4 binding to the putative binding sites of *Il17a* promoter was performed using quantitative RT-PCR with the primers provided in Supplementary Table 3.

### ELISA and ELISPOT

Antibody concentrations in BLF and antibody-producing B cells were monitored throughout the experiment by ELISA and ELISPOT assays, respectively. Total IgM was captured with purified anti-mouse IgM (Biolegend). Anti-influenza antibody was captured with inactivated influenza virus. For ELISA assay, all antibodies were detected with HRP-conjugated secondary anti-mouse IgM (Biolegend). TMB substrate (BioLegend) was used as a substrate for the HRP enzyme. For ELISPOT assay, all antibodies were detected with AP-conjugated anti-mouse IgM antibody (Biolegend). SIGMA*FAST*^TM^ BCIP/NBT (Sigma–Aldrich) was used as a substrate for the AP enzyme.

### Preparation of cardiolipin

Lyophilized CL sodium salt from bovine heart (Sigma–Aldrich) was dissolved in chloroform (CHCl_3_) at 5 mg/mL. Aliquots of dissolved CL or CHCl_3_ alone (vehicle control) were dried down in a glass tube under nitrogen gas. The CL was resuspended in serum-free RPMI1640 medium or PBS, vortexed vigorously for at least 1–2 min and sonicated for 20 min at 37 °C. CL was freshly prepared and vortexed well before use. For in vitro culture experiments, isolated mouse B-1a cells were pulsed with or without 0.2 μM CL in serum-free RPMI1640 for 1 h at 37 °C. After lipid loading, cells were centrifuged and subjected to B-1a-γδ T cell cocultures in 96-well round bottom plates for 3 days at 37 °C. Blocking experiments were performed using 10 μg/mL purified CD1d blocking antibody (1B1, Biolegend). For in vivo transfer, isolated B-1a were cultured with 20 μM CL or vehicle in serum-free RPMI1640 for 1 h at 37 °C. After being washed with PBS, CL or vehicle-pulsed B-1a cells (5 × 10^5^) were injected i.v. into mice. For in vivo injection, pdmH1N1-infected C57BL/6 mice at 1 dpi were treated with a single i.p. injection of CL (100 μg/kg) or vehicle resuspended in 200 μL of PBS.

### Calcium mobilization analysis

For calcium mobilization analysis, single-cell suspensions were harvested from thymus and lung of infected C57BL/6 mice at 4 dpi and were negatively selected to enrich γδ T cells. Briefly, B220^+^, TCRβ^+^, and CD11c^+^ cells were labeled with biotinylated antibodies, magnetically labeled with Anti-Biotin MicroBeads and then negatively depleted over a MACS® Column (Miltenyi Biotec) which was placed in the magnetic field of a MACS Separator. The enriched γδ T cells (>95% purity) were stained with anti-γδTCR-Brilliant Violet 421 and then loaded with 2 μM Fluo-4-AM (Molecular Probes) for 30 min at 37 °C in serum-free medium at a density of 2 × 10^6^ cells/mL. Each sample was heated to 37 °C before analysis. Baseline measurements were achieved by running the samples without stimulation for 30 sec. Dynabeads® Mouse T-Activator CD3/CD28 (Life Techologies) was then added. Data were collected for 600 sec. At 40 sec, ionomycin (500 ng/mL) was added as a calcium-flux control. FlowJo software (TreeStar) was used for data analysis.

### Histology and confocal fluorescence microscopy

Lungs were harvested and immediately frozen in liquid nitrogen and embedded in OCT (Sakuru) for sectioning without further fixation. Lung tissues were cut into 6-μm thin sections using a cryostat microtome/vibratome (Leica). The sections were fixed in ice-cold acetone for 5 min and washed in PBS. Nonspecific binding in tissues was blocked for 30 min with 5% goat serum (Dako) in PBS, followed by washing with PBS and staining for 1 h with the appropriate antibodies. The following antibodies from Biolegend were used for histology examination: anti-mouse CD3ε PE (clone 145-2C11), anti-mouse TCR γ/δ FITC (clone GL3), anti-mouse/human CD45R/B220 PE (clone RA3-6B2), and anti-mouse CD43 APC (clone 1B11). Images were analysed using Carl Zeiss LSM 780 Microscopy and ZEN offline Analysis Software. For histological assessment, lung tissues were placed in 10% neutral buffered formalin for at least 24 h before processing and embedding. Lung tissues were sectioned at 6-μm thickness and stained with hematoxylin and eosin for histopathological evaluation. Slides were examined in a blinded manner and scored with a semi-quantitative system according to the relative degree of inflammation and tissue damage. Briefly, the cumulative scores of inflammatory infiltration, degeneration and necrosis provided the total score per animal. Lung infiltration of inflammatory cells was scored as follows: 0, no inflammation; 1, mild peribronchial and peribronchiolar infiltrates, extending throughout <10% of the lung; 2, moderate inflammation covering 10–50% of the lung; 3, severe inflammation involving over one-half of the lung. Degeneration was scored as follows: 0, no degeneration; 1, little vacuolar degeneration of bronchi and bronchiole epithelium cells, normal pulmonary alveoli; 2, mild necrosis of bronchi and bronchiolar epithelium, mild alveoli damage; 3, severe degeneration. Necrosis was scored as follows: 0, no necrosis; 1, mild necrosis with scant exudate; 2, marked necrosis with abundant exudate; 3, severe interstitial edema around blood vessels, apparent injured parenchyma and degenerated alveolar epithelial cells with greater infiltration of inflammatory cells^6,16^. Images were collected using a light microscope.

### Lipid extraction and derivatization

For lipid extraction from BLF of mice, both cells and insoluble components in the freshly collected BLF samples were removed by high-speed centrifugation at 15,000 × *g* for 20 min. Samples were then dried and resuspended in 1365 μL of methyl tert-butyl ether (MTBE)/Methanol (MeOH) /2 N HCl (v/v/v 200/60/13). The mixtures were incubated at room temperature for 15 min, with 30-second vortex every 5 min. Another 250 μL of 0.1 N HCl was added, followed by centrifugation at 12,000 × *g* for 20 min for phase separation. The upper organic phase was immediately transferred to a new tube and dried by nitrogen gas at room temperature. Dried lipid extracts were resuspended using 400 μL MTBE/MeOH (v/v 200/60). Then 50 μL TMS-diazomethane (2 M in hexane) was added and incubated for 20 min at RT. The reactions were terminated by adding 5 μL of glacial acetic acid and then another 92 μL HPLC-grade water was added. After further centrifugation (12,000 × *g*, 20 min at RT), the upper phase was transferred into a new tube for LC-MS/MS analysis.

### LC-MS/MS method for lipid analysis

Cortecs C18 column (2.1 × 100 mm, Waters) was applied in the analysis. Mobile phase A was made by mixing 400 mL of HPLC-grade water containing 0.77 g of ammonium acetate with 600 mL of HPLC-grade acetonitrile. Mobile phase B contained 10% acetonitrile and 90% isopropanol (v/v). The gradient was as follows: 0 min, 40% B; 3.0 min, 40% B; 23 min, 98% B; 30 min, 98% B; 30.5 min, 40% B; 35 min, 40% B. Data were acquired using Q Exactive orbitrap mass spectrometer (QEHF, Thermo Fisher, USA). Resolution of 60,000 and 30,000 was used for MS and MS/MS acquisition. The detailed mass spectrometer parameters are as follows: spray voltage, 3.2 kV for positive; capillary temperature, 320 °C; aux gas flow rate (arb),10; mass range (*m/z*), 240–2000 for positive mode. Lipids were identified and quantified using Tracefinder 3.2 (Thermo Fisher, USA). In-house database containing 780 methylated CL molecules, which is compatible with Tracerfinder, was applied for lipid identification.

### PRM analysis for targeted cardiolipins

PRM was performed to analyze methylated CL lipids using Q Exactive orbitrap (QEHF, Thermo Fisher, USA) with resolutions of 45,000 in positive ion mode. Eight targeted cardiolipin molecules and one internal standard were included in PRM list with following precursor mass/charge ratios: *m/z* 1449.05300 for CL(34:1/34:2); *m/z* 1475.06865 for CL(34:1/36:3); *m/z* 1473.05300 for CL(34:1/36:4); *m/z* 1473.05300 for CL(34:2/36:3); *m/z* 1469.0217 for CL(34:2/36:5); *m/z* 1469.0217 for CL(34:3/36:4); *m/z* 1499.06865 for CL(36:3/36:3); *m/z* 1495.03735 for CL(36:4/36:4); *m/z* 1286.91105 for internal standard CL(14:0/14:0/14:0/14:0). Isolation window of 2.0 *m/z* was set in the analysis. Stepped normalized collision energy (NCE) of 18, 20, and 22 was used for fragmentation. The parameters of ESI source were as follows: spray voltage, 3.2 kV in positive ion mode; heated capillary temperature, 320 °C; Sheath gas flow rate (Arb), 35; aux gas flow rate (arb), 10. Lipids were identified and quantified using Tracefinder 3.2 (Thermo, CA). The featured fragments of m/z 575.50366, 577.51947, 599.50391, 575.50366, 597.48816, 573.48785, 601.51959 and 599.50391 were applied for quantitation of CL(34:1/34:2), CL(34:1/36:3), CL(34:1/36:4), CL(34:2/36:3), CL(34:2/36:5), CL(34:3/36:4), CL(36:3/36:3), and CL(36:4/36:4).

### Antibiotics treatment and quantification of bacterial load

Mice were exposed to 1% Enrofloxacin (Baytril) ad libitum in drinking water for 13 days. Intranasal administration of 200 μg of Vancomycin (Sigma) and 200 μg of Neomycin (Sigma) in 25 μg of PBS or the equivalent volume of vehicle every other day were performed starting from eight days before pdmH1N1 infection. DNA extractions from BLF and stool samples were done using QIAamp Fast DNA Stool Mini Kit and QIAamp DNA Mini Kit (Qiagen) for detection of pathogens in stool and BLF samples. To perform an absolute quantification, qPCR experiment was performed on a LightCycler 480 (Roche Applied Science) using broad universal primers 63 F and 355 R designed to quantify the 16 S ribosomal DNA gene of the bacteria^74,75^. A standard curve was constructed by amplifying serial dilutions of known quantities of *E. coli* DNA.

### ScRNA-seq and data analysis

Single cells isolated using the methods described in the manuscript were loaded into individual oil droplets following manufacturer’s protocol (10× Genomics®) and processed using the 3’ sequencing kit. The cells were merged into one sample for sequencing via Illumina Hiseq2500. Individually barcoded data were recovered using CellRanger and then loaded into Seurat for initial processing following the standard pipeline. The list of variable genes was used for dimension reduction and UMAP clustering, leading to the identification of the clusters of cells. Functional analyses of differentially expressed genes between the clusters were performed using GSEA, and UMAP visualization of the genesets was performed by summing the contributions of the upregulated factors in each of the genesets identified. Canonical correlation analysis was also performed in Seurat using the standard pipeline. Data on the cells in the Tγδ17 cluster were exported into Monocle for trajectory analyses. Scripts of all of the codes run in this paper are available upon request.

### Statistics

Statistical analysis was performed with GraphPad PRISM 7 software using the two-tailed unpaired Student’s *t*-test for two groups analysis and one-way or two-way ANOVA for multiple groups analysis. Nonparametric data were analysed by Mann–Whitney test. Survival curve of influenza virus-infected mice was determined using Kaplan–Meier analysis. A *P*-value of < 0.05 was considered statistically significant.

### Reporting summary

Further information on research design is available in the Nature Research Reporting Summary linked to this article.

## Supplementary information

Supplementary Information

Reporting Summary

## Data Availability

The single-cell RNA sequencing data have been deposited in GEO under accession code GSE124885 and in NCBI Sequence Read Archive (SRA) under accession code PRJNA644060 for human and mouse γδ T cells, respectively. The datasets generated and analysed during the current study are available from the corresponding authors on reasonable request. The paired analysis of data was performed with existing scRNA-seq data deposited under accession code GSE123400. Source data are provided with this paper.

## Source data

Source Data
