# Supplementary material for: Host-derived lipids orchestrate pulmonary γδ T cell response to provide early protection against influenza virus infection
Source: Nat Commun. 2021 Mar 26;12:1914. doi: 10.1038/s41467-021-22242-9 (PMC7997921; doi:10.1038/s41467-021-22242-9)
Supplement: Supplementary file 1 — Supplementary Information [file 41467_2021_22242_MOESM1_ESM.pdf]

**Supplementary Information**

# Host-derived lipids orchestrate pulmonary γδ T cell response to provide early protection against influenza virus infection

Xiaohui Wang, Xiang Lin, Zihan Zheng, Bingtai Lu, Jun Wang, Andy Hee-Meng Tan, Meng Zhao, Jia Tong Loh, Sze Wai Ng, Qian Chen, Fan Xiao, Enyu Huang, King-Hung Ko, Zhong Huang, Jingyi Li, Kin-Hang Kok, Gen Lu, Xiaohui Liu, Kong-Peng Lam, Wanli Liu, Yuxia Zhang, Kwok-Yung Yuen, Tak Wah Mak and Liwei Lu

Supplementary Fig. 1

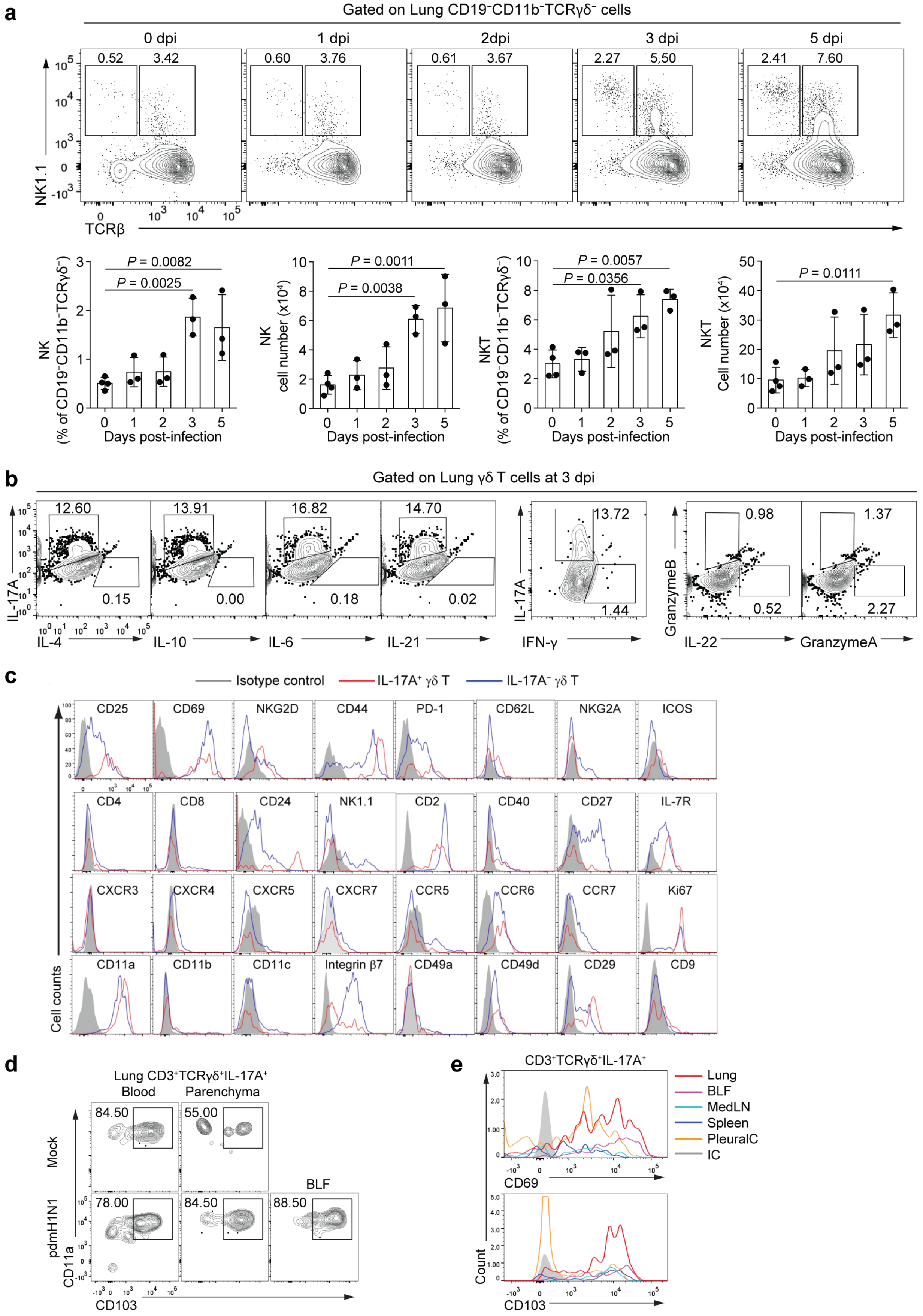


**Supplementary Fig. 1 Innate immunity against pdmH1N1 infection. a**, Representative flow cytometric plots (upper) and frequency and cell number (lower) of NK and NKT cells in pdmH1N1-infected lungs of C57BL/6 mice (n = 4, 3, 3, 3). Data are combined from three independent experiments and presented as mean ± SD. *P* values were determined using one-way ANOVA. **b**, Flow cytometry analysis of cytokine

production by lung γδ T cells from pdmH1N1-infected mice at 3 dpi. Data were representative of three independent experiments. **c**, Expression of cell-surface or intranuclear molecules on lung IL-17A$^+$ γδ T (red line) and IL-17A$^-$ γδ T (blue line) from pdmH1N1-infected mice at 5dpi. Shaded histograms depict staining by isotype control (IC) antibody. Data were representative of three independent experiments. **d**, Flow cytometry plots showing the expression of CD103 and CD11a on lung circulating (CD45$^+$) and parenchyma-associated (CD45$^-$) CD3$^+$γδTCR$^+$IL-17A$^+$ cells in mock-infected or pdmH1N1-infected mice, and CD45$^-$ CD3$^+$TCRγδ$^+$IL-17A$^+$ cells in BLF of infected mice. **e**, Flow cytometry analysis of the expression of CD69 and CD103 on CD3$^+$TCRγδ$^+$IL17A$^+$ cells in lung, BLF, MedLN, spleen, pleural cavity of infected mice at 5dpi. Source data are included in Source Data file.

Supplementary Fig. 2

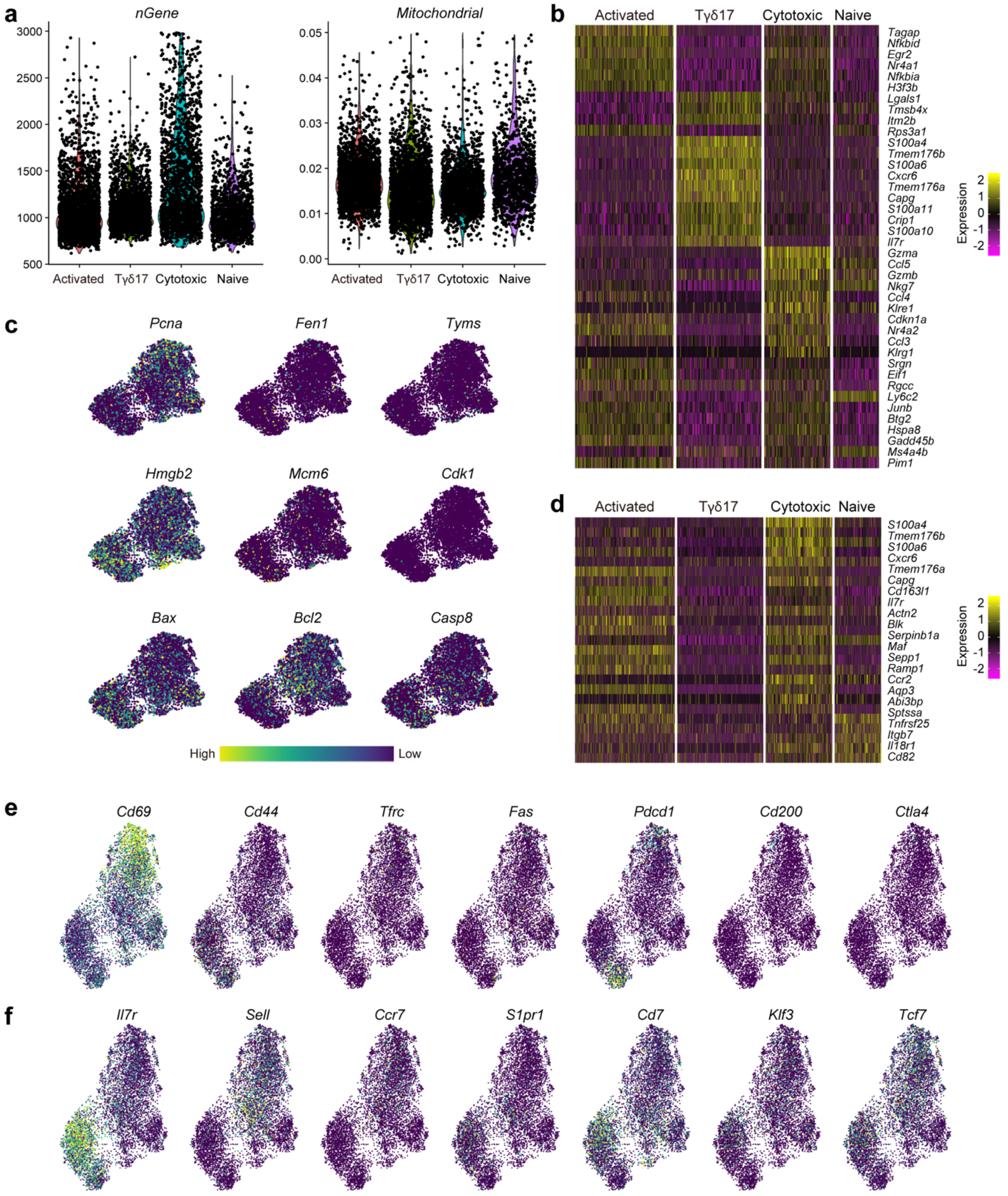


**Supplementary Fig. 2 Justification for clustering of lung γδ T cells. a**, Violin plot following filtering based on mitochondrial gene percentage (4%) and the total number of genes detected (between 300 and 2500) and reduced the total amount of cells considered to 7,863 cells from a starting pool of over 9,000 cells. **b,** Heatmap visualization of the expression patterns of the most varied genes in each cluster across each cell in the cluster. These highly varied genes were used to help annotate the clusters. **c,** UMAP visualization of the expression levels of prominent genes associated with T cell proliferation and cell cycle. **d,** Heatmap visualization of the expression patterns of the most varied genes in each cluster across each cell in the cluster. These highly varied genes were used to help annotate the clusters. **e,f,** UMAP visualization of the expression levels of prominent genes associated with T cell activation (**e**) and differentiation (**f**).

Supplementary Fig. 3

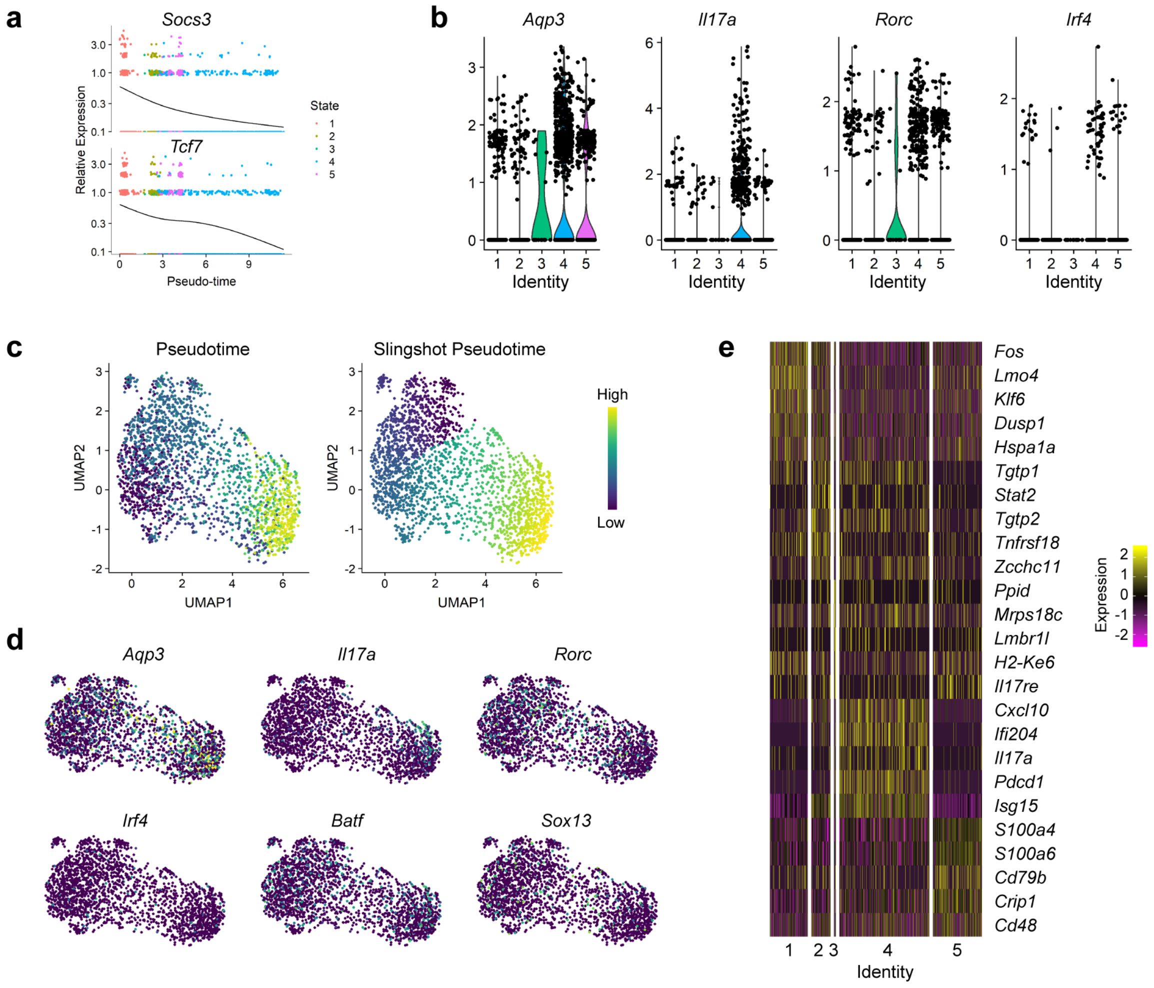


**Supplementary Fig. 3 *Aqp3* and *Irf4* expression identifies Tγδ17 subset. a**, Pseudotime mapping of the expression levels of *Socs3* and *Tcf7* shows a clear decrease over pseudotime that likely renders the cells more permissive to maturation. **b**, Violin plots of the expression profiles of *Il17a*-related genes as shown in Fig. 4e. **c**, UMAP visualization performed in Seurat of the Tγδ17 cluster following assignment of state and alignment according to pseudotime in Monocle. **d**, UMAP visualization of the expression profiles for key genes. **e**, Heatmap visualization of the expression patterns of the most varied genes in each cluster across each cell in the cluster. These highly varied genes were used to help annotate the clusters.

Supplementary Fig. 4

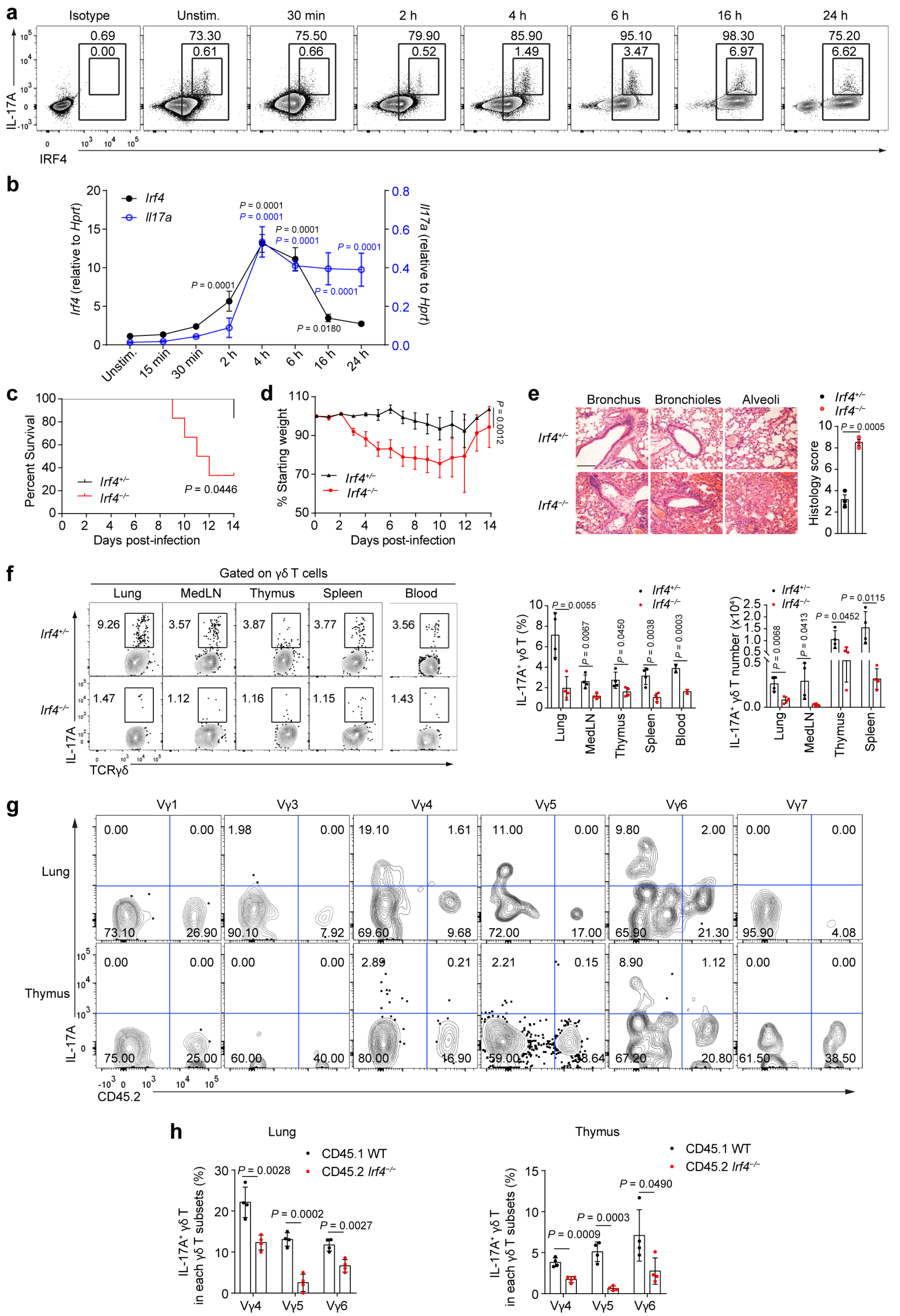

**Supplementary Fig. 4 IRF4 is required for IL-17A production in γδ T cells. a,b**, Purified γδ T cells were stimulated for the indicated lengths of time with soluble anti-CD3/CD28 beads (5 μg/mL). IRF4 and IL-17A

induction and their transcripts expression were measured by flow cytometry (**a**) and quantitative PCR (**b**) (n = 3). One-way ANOVA. **c-e,** The Kaplan-Meier survival curve (**c**, n = 6, Gehan-Breslow-Wilcoxon Test) and body weight (**d**, n = 6, two-way ANOVA test) of heterozygous IRF4 ($Irf4^{+/-}$) and $Irf4^{-/-}$ mice infected with pdmH1N1 were monitored for 14 successive days. (**e**) H&E histology (left) and scoring (right) of lungs from mice at 5 dpi (n = 3). Two-tailed unpaired Student's t test. Images are at original magnification x 200. Scale bar, 40 μm. **f,** $Irf4^{+/-}$ and $Irf4^{-/-}$ mice were challenged with pdmH1N1 and analysed by flow cytometry at 5 dpi. Flow cytometry plots (left) and cumulative data (right) showing frequencies (n = 4, 4, 4, 4, 3) and numbers (n = 4 per group) of IL-17A$^+$ cells in gated CD3$^+$TCRγδ$^+$ cells from the indicated organs. Two-tailed unpaired Student's t test. **g,h**, Bone marrow chimeric mice generated with wild-type (CD45.1$^+$) and IRF4 deficient ($Irf4^{-/-}$) (CD45.2$^+$) bone marrow cells were challenged with pdmH1N1 and analysed by flow cytometry at 5 dpi. (**g**) The IL-17A expression in gated Vγ1/3/4/5/6/7$^+$CD3$^+$TCRγδ$^+$ live singlets from lung and thymus was examined by flow cytometry. (**h**) Cumulative data showing frequencies of IL-17A$^+$ cells in CD45.1$^+$/ CD45.2$^+$ Vγ4/5/6$^+$CD3$^+$TCRγδ$^+$ cells from lung and thymus (*n* = 4). Two-tailed unpaired Student's t test. Data are combined from two or three independent experiments and presented as mean ± SEM. Source data are included in Source Data file.

Supplementary Fig. 5

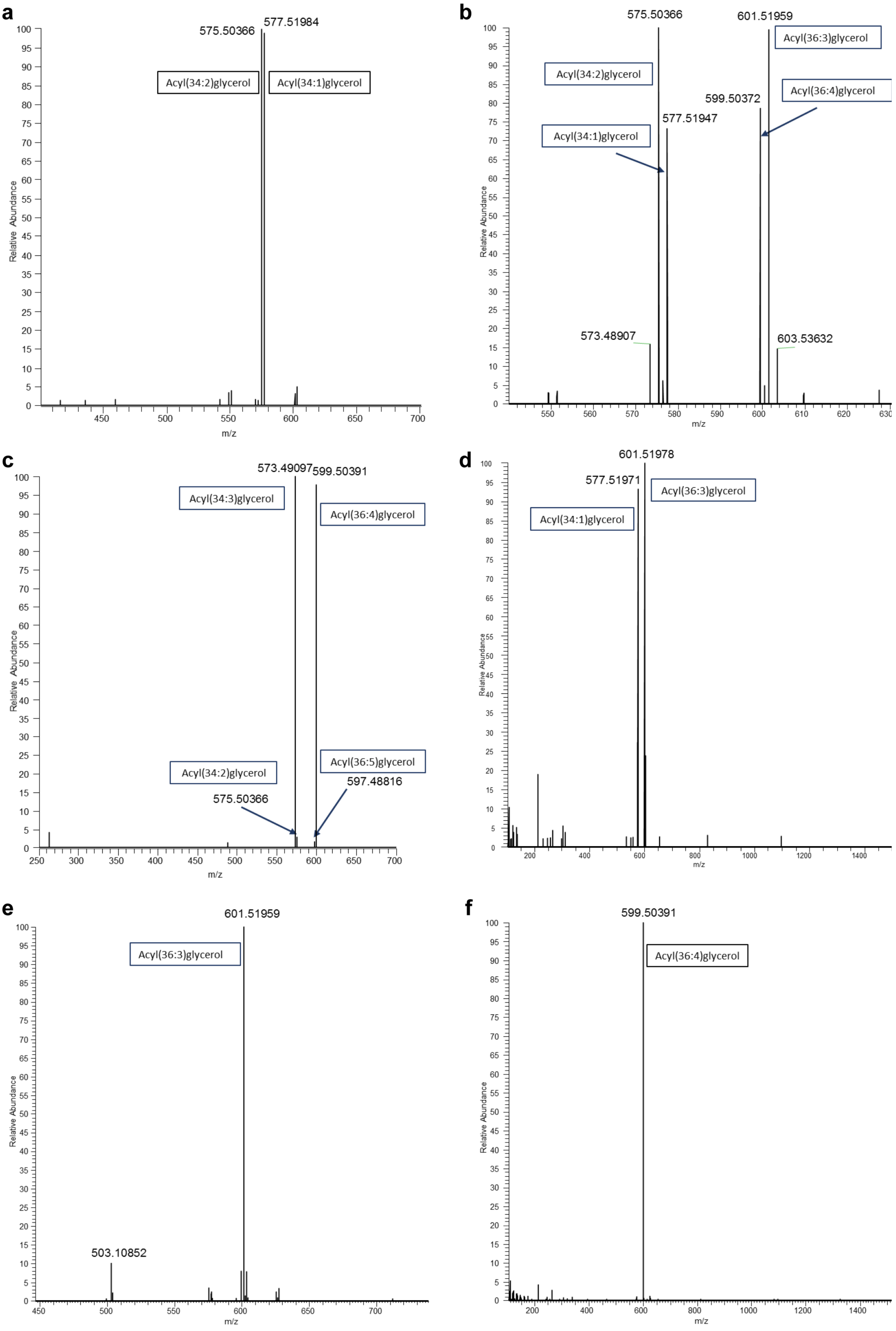

**Supplementary Fig. 5 MS/MS spectra used for CL identification.** Representative MS/MS spectra of (**a**) CL(34:1/34:2), (**b**) mixed CL(34:1/36:4) and CL(34:2/36:3), (**c**) mixed CL(34:2/36:5) and CL(34:3/36:4), (**d**) CL(34:1/36:3), (**e**) CL(36:3/36:3) and (**f**) CL(36:4/36:4) are shown. (**b**) and (**c**) showed the mixed fragments from isomeric CL lipid molecules. Selected mass range was displayed for better visualization of featured fragments (n = 5).

Supplementary Fig. 6

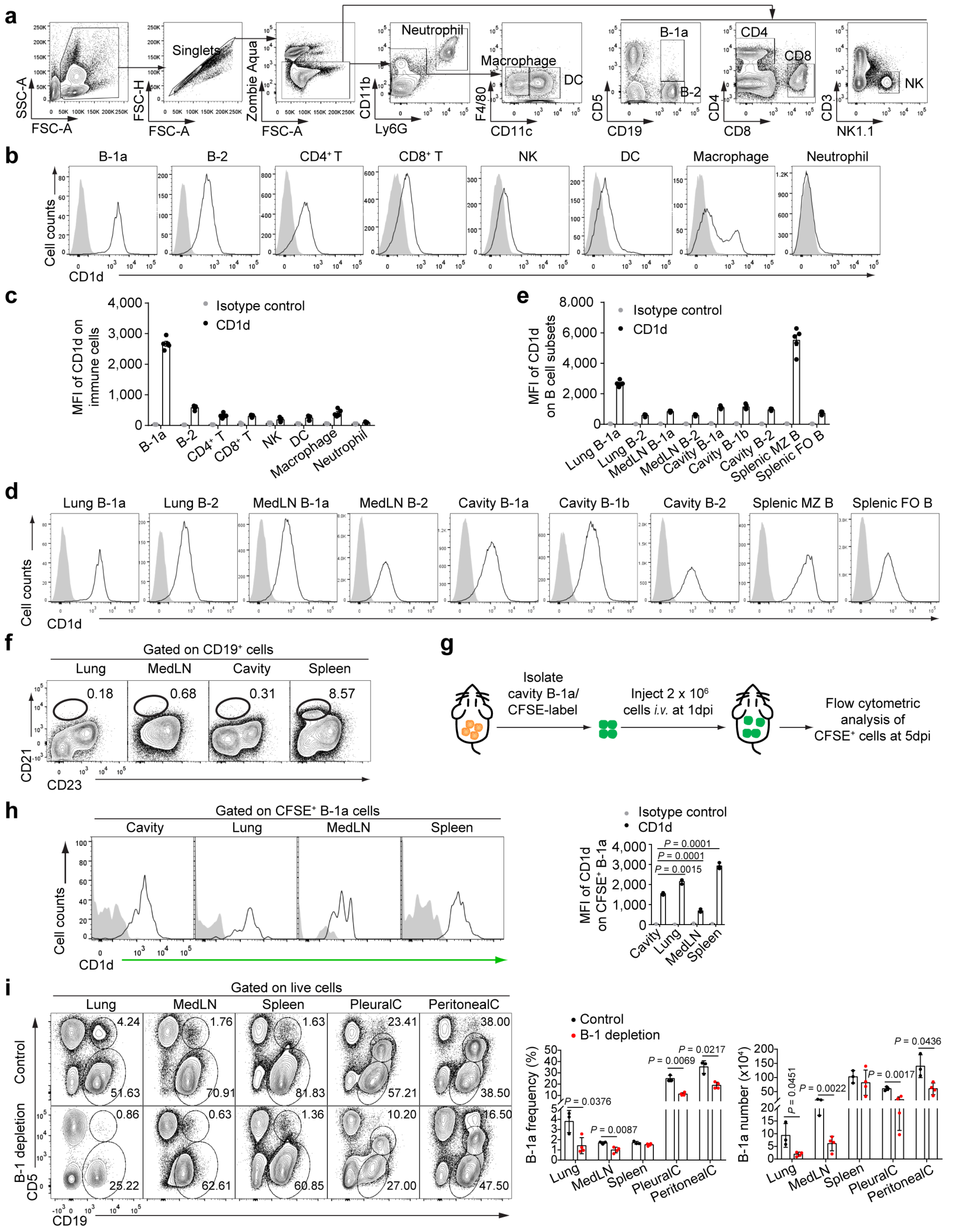


**Supplementary Fig. 6 Lung B-1a cells upregulate CD1d expression during pdmH1N1 infection. a**, Gating strategy for immune cell populations in the lungs from pdmH1N1-infected C57BL/6 mice at 3 dpi. **b**,**c**, Flow cytometric analysis (**b**) of CD1d expression (black lines) on immune cell populations in the lungs from infected mice at 3 dpi. Shaded histograms depict staining by isotype control antibody. Cumulative data (**c**) showing MFI of CD1d expression ($n$ = 5). **d**,**e**, Flow cytometry analysis (**d**) showing CD1d expression (black lines) on B cell subsets in the lung, MedLN, pleural cavity and spleen from C57BL/6 mice at 3 dpi.

Shaded histograms depict staining by isotype control antibody. Cumulative data (**e**) showing MFI of CD1d expression. MZ B, marginal zone B cell; FO B, follicular B cell (*n* = 5). **f**, Flow cytometry analysis of MZ B cells in the indicated organs from C57BL/6 mice at 3 dpi. **g,h**, Flow chart (**g**) of B-1a cell labelling, transfer and analysis. Flow cytometric analysis (**h**, left) of CD1d expression (black lines) on $CFSE^+$ B-1a cells from the indicated organs. Shaded histograms depict staining by isotype control antibody. Cumulative data on the right showing MFI of CD1d expression (n = 3). **i**, B-1 cell depletion was achieved as described in Fig. 6c. Representative flow cytometry plots (left) and cumulative data (right) showing frequencies and cell numbers of B-1a cells in the indicated organs at 4dpi (*n* = 3, 4). Data are combined from two independent experiments and presented as mean ± SEM. *P* values were determined using two-tailed unpaired Student's t test (**h, i**). Source data are included in Source Data file.

Supplementary Fig. 7

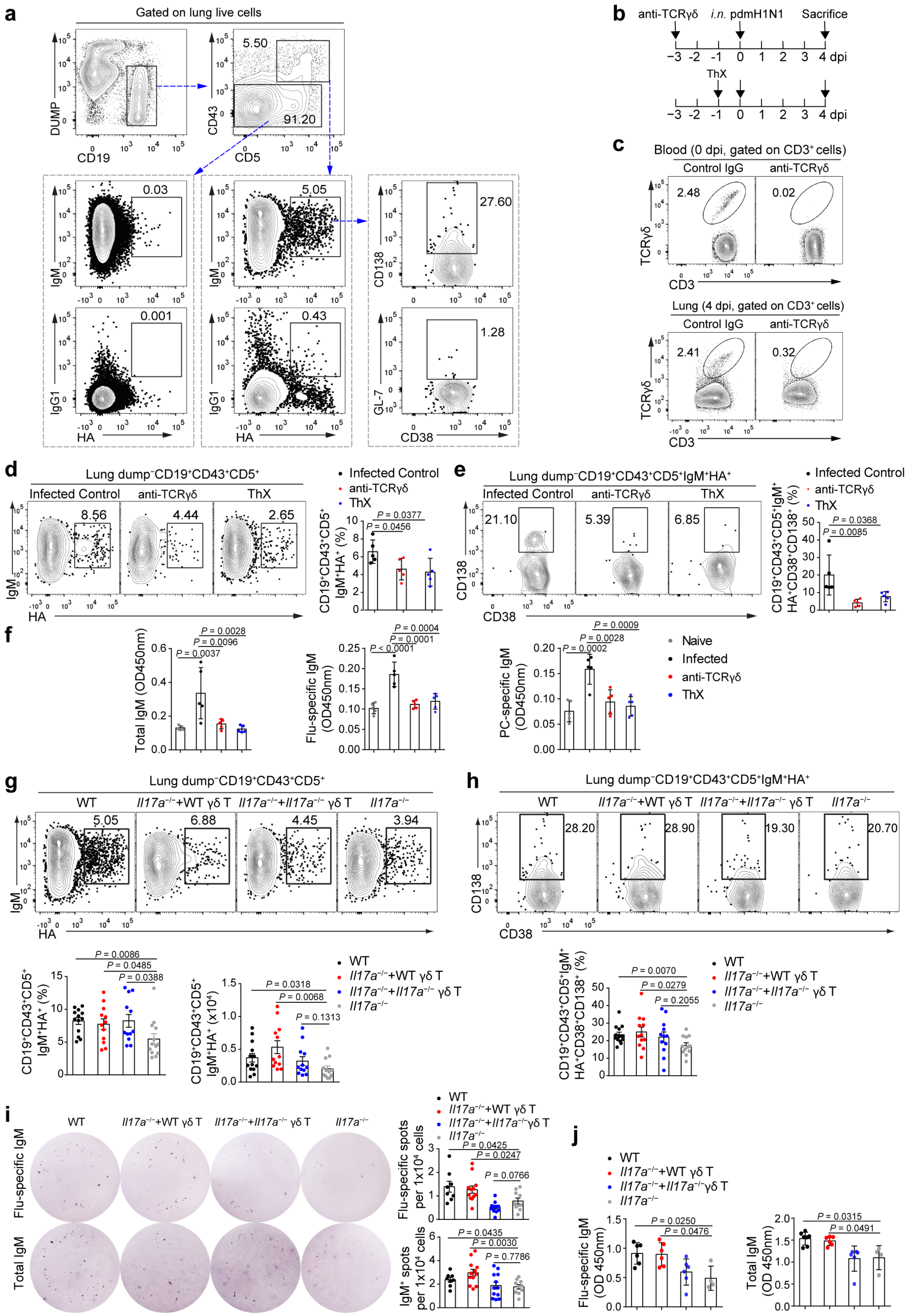

**Supplementary Fig. 7 Tγδ17 cells promote plasmacytic differentiation of B-1a cells. a**, Flow cytometry analysis of lung HA-specific B cells from pdmH1N1-infected mice at 4 dpi, gating on the dump-negative

(CD3$^-$CD11c$^-$Gr1$^-$NK1.1$^-$F4/80$^-$TER$^-$Live/Dead-Aqua$^-$) CD19$^+$ fraction. **b**, Schema of γδ T cell targeting and ThX surgery in (**c-f**). Mice received a single *i.p.* injection of 200 μg anti-TCRγδ antibody three days before infection (upper). Another group of mice received ThX one day before infection (lower). **c**, Representative plots showing the efficacy and specificity of γδ T cell silencing in the blood at 0 dpi and lung from infected mice at 4 dpi (n = 5). **d**, Flow cytometry analysis (left) and cumulative data (right) showing frequencies of IgM$^+$HA$^+$ cells in gated lung dump$^-$CD19$^+$CD43$^+$CD5$^+$ cells (n = 5). **e**, Flow cytometry analysis (left) and cumulative data (right) showing frequencies of CD38$^+$CD138$^+$ B cells in gated lung dump$^-$CD19$^+$CD43$^+$CD5$^+$IgM$^+$HA$^+$ cells (n = 5). **f**, Total, influenza virus-specific and phosphorylcholine (PC)-specific IgM in BLF were determined by ELISA assay (n = 5). **g-j**, PdmH1N1-infected *Il17a*$^{-/-}$ mice at 1 dpi were *i.v.* transferred with 2 x $10^6$ γδ T cells purified from WT or *Il17a*$^{-/-}$ mice at 4 dpi and analysed at 4 dpi (n = 13). **g**, Flow cytometry analysis and cumulative data (below) showing IgM$^+$HA$^+$ cells in gated lung dump$^-$CD19$^+$CD43$^+$CD5$^+$ cells (n = 13, 12, 12, 13). **h**, Flow cytometry analysis and cumulative data (below) showing CD38$^+$CD138$^+$ cells in gated lung dump$^-$CD19$^+$CD43$^+$CD5$^+$IgM$^+$HA$^+$ cells (n = 13, 12, 12, 13). **i**, Virus-specific IgM and total IgM-producing cells in lung cells were measured by ELISPOT at 4 dpi (left). Cumulative data (right) showing numbers of spot-forming cells per $10^4$ input cells (n = 8, 12, 12, 10) . **j**, Virus-specific IgM (n = 6, 6, 6, 4) total IgM (n = 7, 6, 6, 4) in serum at 5 dpi were examined with ELISA. Data are combined from two or three independent experiments and are represented as mean ± SEM. *P* values were determined using two-tailed unpaired Student's t test (**g, h, i**), or one-way ANOVA (**d, e, f, j**). Source data are included in Source Data file.

Supplementary Fig. 8

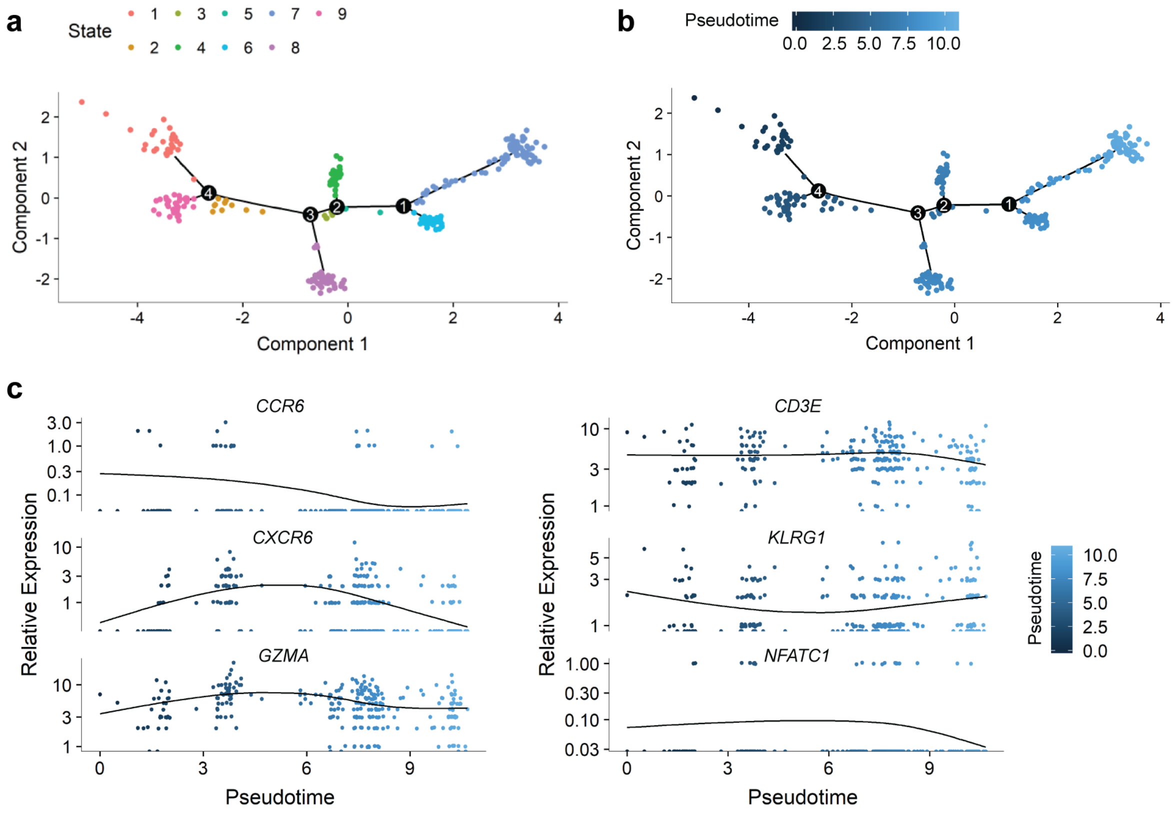


**Supplementary Fig. 8 Human lung γδ T cells exhibit conserved molecular signature. a,b**, Trajectory map generated using DDTree reduction as implemented through Monocle on a list of 1,224 genes with detectable expression and significant dispersion within γδ T cells. The computed pseudotime overlaid on the trajectory map suggests that 2 distinct starting states lead to 2 different end states for the Tγδ17 subset. **c,** Pseudotime mapping of the expression levels of *CCR6, CXCR6, GZMA, CD3E, KLRG1* and *NFATC1* shows a clear decrease over pseudotime that likely renders the cells more permissive to maturation.

**Supplementary Table 1. Cohort characteristics of human participants.**

| | Moderate group | Severe group | *p* values* |
|---|---|---|---|
| Number | 56 | 42 | |
| Demographics | | | |
| Gender (M/F), (%) | 40 (71.4%)/16 (28.6%) | 34 (80.9%)/8 (19.1%) | n.s.# |
| Age (Months), Median (Range) | 36, (2-156) | 36, (5-144) | n.s. |
| Body weight (Kg), Median (Range) | 14.0, (6.2-39.0) | 13.8, (6.3-44.9) | n.s. |
| Hospital stay (Days), Median (Range) | 1, (1-34) | 12, (1-18) | 0.0008 |
| Infection types, No. (%) | | | |
| Viral infections or only | 20 (35.7%) | 30 (71.4%) | |
| Bacterial infections only | 4 (7.1%) | 1 (2.4%) | |
| Viral+ Bacterial infections | 8 (14.3%) | 6 (14.3%) | |
| No pathogen detected | 23 (41.1%) | 1 (2.4%) | |
| No record | 1 (1.8%) | 4 (9.5%) | |

*: *p* values were calculated by unpaired *t* test.

#: *p* values were calculated by Fisher's exact test.

## Supplementary Table 2. Antibody list.

| Reagent | Source | Identifier |
|---|---|---|
| **Antibodies (FACS)** | | |
| Anti-mouse CD3 PE/Cy7 (145-2C11) | Biolegend | Cat# 100320 |
| Anti-mouse γδTCR Brilliant Violet 421™ (GL3) | Biolegend | Cat# 118120 |
| Anti-mouse CD69 PerCP/Cy5.5 (H1.2F3) | Biolegend | Cat# 104522 |
| Anti-mouse CD25 PE/Cy5 (PC61) | Biolegend | Cat# 102010 |
| Anti-mouse NKG2D PE (CX5) | Biolegend | Cat# 130208 |
| Anti-mouse NKG2A APC (16A11) | Biolegend | Cat# 142808 |
| Anti-mouse/human CD44 PE/Cy7 (IM7) | Biolegend | Cat# 103030 |
| Anti-mouse PD-1 APC (29F.1A12) | Biolegend | Cat# 135210 |
| Anti-mouse CD62L PE/Cy5 (MEL-14) | Biolegend | Cat# 104410 |
| Anti-mouse CD45 FITC (30-F11) | Biolegend | Cat# 103108 |
| Anti-mouse CD45.2 APC (104) | Biolegend | Cat# 109814 |
| Anti-mouse ICOS PE (7E.17G9) | Biolegend | Cat# 117406 |
| Anti-mouse CD4 PE/Cy5 (RM4-5) | Biolegend | Cat# 100514 |
| Anti-mouse CD8 PerCP/Cy5.5 (53-6.7) | Biolegend | Cat# 100734 |
| Anti-mouse CD24 PE/Cy5 (M1/69) | Biolegend | Cat# 101808 |
| Anti-mouse CD1d PerCP/Cy5.5 (1B1) | Biolegend | Cat# 123514 |
| Anti-mouse NK1.1 FITC (PK136) | Biolegend | Cat# 108706 |
| Anti-mouse CD40 PE/Cy5 (3/23) | Biolegend | Cat# 124618 |
| Anti-mouse/rat/human CD27 PE/Cy7(LG.3A10) | Biolegend | Cat# 124216 |
| Anti-mouse CXCR3 PE(CXCR3-173) | Biolegend | Cat# 126506 |
| Anti-mouse CXCR4 PerCP/Cy5.5 (L276F12) | Biolegend | Cat# 146510 |
| Anti-mouse CXCR5 PE (L138D7) | Biolegend | Cat# 145504 |
| Anti-mouse/human CXCR7 PE/Cy7 (8F11-M16) | Biolegend | Cat# 331116 |
| Anti-mouse CCR6 APC (29-2L17) | Biolegend | Cat# 129814 |
| Anti-mouse CCR5 PE (HM-CCR5) | Biolegend | Cat# 107006 |
| Anti-mouse CCR6 APC (29-2L17) | Biolegend | Cat# 129814 |
| Anti-mouse CCR7 APC (4B12) | Biolegend | Cat# 120108 |
| Anti-mouse CD11a PE/Cy7 (M17/4) | Biolegend | Cat# 101122 |
| Anti-mouse/human CD11b PE/Cy5 (M1/70) | Biolegend | Cat# 101210 |
| Anti-mouse mouse CD11c PE/Cy7 (N418) | Biolegend | Cat# 117318 |
| Anti-mouse CD49a PE (HMα1) | Biolegend | Cat# 142604 |
| Anti-mouse CD49d PE (9C10(MFR4.B)) | Biolegend | Cat# 103706 |
| Purified anti-mouse CD16/32 (93) | Biolegend | Cat# 101302 |
| Anti-mouse CD29 FITC (HMβ1-1) | Biolegend | Cat# 102206 |
| Anti-mouse CD9 FITC (MZ3) | Biolegend | Cat# 124808 |
| Anti-human/mouse Integrin β7 FITC (FIB504) | Biolegend | Cat# 321214 |
| Anti-mouse CD19 Brilliant Violet 421™ (6D5) | Biolegend | Cat# 115538 |
| Anti-mouse CD43 PE/Cy5 (1B11) | Biolegend | Cat# 121216 |
| Anti-mouse CD38 APC/Cy7 (90) | Biolegend | Cat# 102728 |
| Anti-mouse CD5 APC (53-7.3) | Biolegend | Cat# 100626 |
| Anti-mouse CD138 PE (281-2) | Biolegend | Cat# 142504 |
| Anti-mouse/human GL7 PerCP/Cy5.5 (GL7) | Biolegend | Cat# 144610 |
| Anti-mouse IgG1 PE-CF594 (A85-1) | BD Biosciences | Cat# 562559 |
| Anti-mouse IgM Brilliant Violet 421™ (RMM-1) | Biolegend | Cat# 406518 |
| Anti-mouse Ly-6G/Ly-6C (Gr-1) FITC (RB6-8C5) | Biolegend | Cat# 108406 |
| Anti-mouse Ly6G APC (1A8) | Biolegend | Cat# 127614 |

| | | |
|---|---|---|
| Anti-mouse F4/80 PerCP/Cy5.5 (BM8) | Biolegend | Cat# 123128 |
| Anti-mouse TER-119 FITC (Ly-76) | Biolegend | Cat# 116206 |
| Anti-mouse TCR Vγ1.1 FITC (2.11) | Biolegend | Cat# 141104 |
| Anti-mouse TCR Vγ3 PE (536) | Biolegend | Cat# 137504 |
| Anti-mouse TCR Vγ4 APC (UC3-10A6) | Biolegend | Cat# 137708 |
| Anti-mouse TCR Vγ7 (GL1.7) | Gift from Dr. Rebecca L. O'Brien (National Jewish Health, Denver, CO) | |
| Anti-mouse TCR Vγ5Vδ1 (17D1) | Gift from Dr. Robert E Tigelaar and Dr. Julia M. Lewis (Yale School of Medicine, Connecticut, USA) | |
| Anti-mouse TCR Vγ6Vδ1 (GL3+17D1) | Gift from Dr. Robert E Tigelaar and Dr. Julia M. Lewis (Yale School of Medicine, Connecticut, USA) | |
| Purified anti-mouse TCR γ/δ (GL3) | Biolegend | Cat# 118101 |
| Anti-rat IgM FITC (MRM-47) | Biolegend | Cat# 408905 |
| Anti-human CD20 Brilliant Violet 421™ (2H7) | Biolegend | Cat# 302330 |
| Anti-human CD19 APC/Cy7 (HIB19) | Biolegend | Cat# 302218 |
| Anti-mouse/rat/human CD27 PE/Cy7 (LG.3A10) | Biolegend | Cat# 124216 |
| Anti-human CD43 APC (CD43-10G7) | Biolegend | Cat# 343206 |
| Anti-human CD70 FITC (113-16) | Biolegend | Cat# 355106 |
| Anti-human CD1d PE (51.1) | Biolegend | Cat# 350306 |
| Antihuman CD3 PE/Cy7 (HIT3a) | Biolegend | Cat# 300316 |
| Anti- human TCR γ/δ Brilliant Violet 421™ (B1) | Biolegend | Cat# 331218 |
| Anti-human TCR Vγ9 APC (B3) | Biolegend | Cat# 331310 |
| Anti-human IL-17A APC (BL168) | Biolegend | Cat# 512334 |
| Anti-mouse IL-17A PE (TC11-18H10.1) | Biolegend | Cat# 506904 |
| Anti-mouse Granzyme A PE (3G8.5) | Biolegend | Cat# 149704 |
| Anti-human/mouse Granzyme B (GB11) | Biolegend | Cat# 515403 |
| Anti-mouse IL-4 PE (11B11) | Biolegend | Cat# 504104 |
| Anti-mouse IL-6 APC (MP5-20F3) | Biolegend | Cat# 504508 |
| Anti-mouse IL-10 APC (JES5-16E3) | BD Biosciences | Cat# 554468 |
| Anti-mouse IL-21 PE (mhalx21) | eBioscience | Cat# 12-7213-80 |
| Anti-mouse IL-22 PE (Poly5164) | Biolegend | Cat# 516404 |
| Anti-mouse IFN-γ PE (XMG1.2) | eBioscience | Cat# 12-7311-41 |
| Anti-human/mouse PE/Cy7 (3E4) | eBioscience | Cat# 25-9858-82 |
| **Antibodies *(In vivo* depletion)** | | |
| Purified anti-mouse IgM (RMM-1) | Biolegend | Cat# 406502 |
| LEAF™ Purified anti-mouse TCR γ/δ (GL3) | Biolegend | Cat# 118114 |
| **Antibodies *(In vivo* and *in vitro* blocking)** | | |
| LEAF™ Purified anti-mouse CD1d (1B1) | Biolegend | Cat# 123504 |
| LEAF™ Purified anti-human CD1d (51.1) | Biolegend | Cat# 350304 |
| **Antibodies (Histology)** | | |
| Anti-mouse CD3ε PE (145-2C11) | Biolegend | Cat# 100308 |
| Anti-mouse TCR γ/δ FITC (GL3) | Biolegend | Cat# 118106 |
| Anti-mouse/human CD45R/B220 PE (RA3-6B2) | Biolegend | Cat# 103208 |
| Anti-mouse CD43 APC (1B11) | Biolegend | Cat# 121214 |
| **Antibodies (ELISA)** | | |
| Purified anti-mouse IgM (RMM-1) | Biolegend | Cat# 406502 |
| Anti-mouse IgM Biotin (RMM-1) | Biolegend | Cat# 406504 |
| **Antibodies (CHIP)** | | |
| Anti-human/mouse/rat IRF-4 (D9P5H) | Cell Signaling | Cat# 15106 |

**Supplementary Table 3. Oligonucleotides.**

| Oligonucleotides | DNA sequences |
|---|---|
| *Il17a* primer 1 (137-146bp) | Forward:ACAGGTCAAGAGAAACAGACAGCCACAT; |
| ChIP real-time PCR | Reverse:CAGTCAGTACTCTTAACTGCTGAGCCATCT |
| *Il17a* primer 2 (248-266bp) | Forward:CAAGAGAAACAGACAGCCACATACCAAAGA; |
| ChIP real-time PCR | Reverse: CACCATGTGGTTTCTGGGAATTGAACTCA |
| *Il17a* primer 3 (436-439bp) | Forward:TCCTACAAGTGTGCTGATAAACAGGGTGAA; |
| ChIP real-time PCR | Reverse:CCTTCTAGCCTCCATGTTACTATGCTCCAT |
| *Il17a* primer 4 (559-562bp) | Forward:TCCTACAAGTGTGCTGATAAACAGGGTGAA; |
| ChIP real-time PCR | Reverse:CCTTCTAGCCTCCATGTTACTATGCTCCAT |
| *Il17a* primer 5 (625-628bp) | Forward:TCCTACAAGTGTGCTGATAAACAGGGTGAA; |
| ChIP real-time PCR | Reverse:CCTTCTAGCCTCCATGTTACTATGCTCCAT |
| *Il17a* primer 6 (750-753bp) | Forward:TTCAAGGAGTTCATGCTTCTCATTCAGGAA; |
| ChIP real-time PCR | Reverse:GACACTTCTTGGGATACCCTTGGGATAATAC |
| *Il17a* primer 7 (836-842bp) | Forward:ATCCCAAGGGTATCCCAAGAAGTGTCAG; |
| ChIP real-time PCR | Reverse:TGTCTGGTAGGATTGCTGGATTCTGTTCA |
| *Il17a* primer 8 (843-948bp) | Forward:ATCCCAAGGGTATCCCAAGAAGTGTCAG; |
| ChIP real-time PCR | Reverse:TGTCTGGTAGGATTGCTGGATTCTGTTCA |
| *Il17a* primer 9 (1099-1102bp) | Forward:CTTGTCCTCATATCTGCTATTCCTGAAGAA; |
| ChIP real-time PCR | Reverse:GATGAAGGCTGAAAGACAGTGGAGAC |
| *Il17a* primer 10 (1333-1336bp) | Forward:TCAGTCCCTTATTCTTTCACTTCATTTCCTTCCTC; |
| ChIP real-time PCR | Reverse:AGGCTAGTTAGTTATTAAGGAGTAGCGAGAGACAT |
| *Il17a* primer 11 (1400-1407bp) | Forward:TCAGTCCCTTATTCTTTCACTTCATTTCCTTCCTC; |
| ChIP real-time PCR | Reverse:AGGCTAGTTAGTTATTAAGGAGTAGCGAGAGACAT |
| *Il17a* primer 12 (1485-1493bp) | Forward:CTAGTAAACCTCATGTCTCTCGCTACTCCTTAA; |
| ChIP real-time PCR | Reverse:CCCTTGCCCAAAGAAACCCACTCAATG |
| *Il17a* primer 13 (1629-1641bp) | Forward:TGAGTGGGTTTCTTTGGGCAAGGGATG; |
| ChIP real-time PCR | Reverse:TGTGAGTCTCTTGACAGTCTCCTAGTGACA |
| *Il17a* primer 14 (1757-1763bp) | Forward:TGTCACTAGGAGACTGTCAAGAGACTCACAA; |
| ChIP real-time PCR | Reverse:GAAGGTAGATGGGAAGGGCAGAAGTTCAC |
| *Il17a* primer 15 (1881-1888bp) | Forward:TGAACTTCTGCCCTTCCCATCTACCTT; |
| ChIP real-time PCR | Reverse:ACTACCTCTGTGGTCACTTACGTCAAGA |
| *Il17a* primer 16 (1982-1985bp) | Forward:CGTAAGTGACCACAGAGGTAGTAA; |
| ChIP real-time PCR | Reverse:GCAGGACTCACCACAGATGAA |
