# Supplementary material for: Host-derived lipids orchestrate pulmonary γδ T cell response to provide early protection against influenza virus infection
Source: Nat Commun. 2021 Mar 26;12:1914. doi: 10.1038/s41467-021-22242-9 (PMC7997921; doi:10.1038/s41467-021-22242-9)
Supplement: Supplementary file 2 — Reporting Summary [file 41467_2021_22242_MOESM2_ESM.pdf]

nature research

Corresponding author(s): Liwei Lu

Last updated by author(s): Feb 25, 2021

Reporting Summary

Nature Research wishes to improve the reproducibility of the work that we publish. This form provides structure for consistency and transparency in reporting. For further information on Nature Research policies, see Authors & Referees and the Editorial Policy Checklist .

Statistics

For all statistical analyses, confirm that the following items are present in the figure legend, table legend, main text, or Methods section.

n/a	Confirmed	
☐	☒	The exact sample size (*n*) for each experimental group/condition, given as a discrete number and unit of measurement
☐	☒	A statement on whether measurements were taken from distinct samples or whether the same sample was measured repeatedly
☐	☒	The statistical test(s) used AND whether they are one- or two-sided<br>*Only common tests should be described solely by name; describe more complex techniques in the Methods section.*
☐	☒	A description of all covariates tested
☐	☒	A description of any assumptions or corrections, such as tests of normality and adjustment for multiple comparisons
☐	☒	A full description of the statistical parameters including central tendency (e.g. means) or other basic estimates (e.g. regression coefficient) AND variation (e.g. standard deviation) or associated estimates of uncertainty (e.g. confidence intervals)
☐	☒	For null hypothesis testing, the test statistic (e.g. *F*, *t*, *r*) with confidence intervals, effect sizes, degrees of freedom and *P* value noted<br>*Give P values as exact values whenever suitable.*
☒	☐	For Bayesian analysis, information on the choice of priors and Markov chain Monte Carlo settings
☒	☐	For hierarchical and complex designs, identification of the appropriate level for tests and full reporting of outcomes
☒	☐	Estimates of effect sizes (e.g. Cohen's *d*, Pearson's *r*), indicating how they were calculated

Our web collection on statistics for biologists contains articles on many of the points above.

Software and code

Policy information about availability of computer code

Data collection	Flow cytometry data were acquired on a BD FACSAria SORP (Becton Dickinson) and analysed using FlowJo software (Version 10, TreeStar); Confocal images were acquired by Carl Zeiss LSM 800; LC/MS data were acquired using QExactive orbitrap mass spectrometer; Single cell sequencing was performed using Illumina Hiseq2500, and individually barcoded data were recovered using CellRanger and then loaded into Seurat for initial processing following the standard pipeline.
Data analysis	Analysis of single cell sequencing data was performed using GSEA, UMAP and Seurat as mentioned in the Methods section. Confocal images were analyzed by Zen 2.3 Blue Edition. All other analysis was performed using GraphPad PRISM 7 software as mentioned in the Methods section. Code availability: The single-cell RNA sequencing data have been deposited in GEO under accession number GSE124885 (https://www.ncbi.nlm.nih.gov/geo/query/acc.cgi?acc=GSE124885) and in NCBI Sequence Read Archive (SRA) under accession number PRJNA644060 (https://www.ncbi.nlm.nih.gov/sra/?term=PRJNA644060) on human and mouse γδ T cells, respectively.

For manuscripts utilizing custom algorithms or software that are central to the research but not yet described in published literature, software must be made available to editors/reviewers. We strongly encourage code deposition in a community repository (e.g. GitHub). See the Nature Research guidelines for submitting code & software for further information.

Data

Policy information about availability of data

All manuscripts must include a data availability statement. This statement should provide the following information, where applicable:

- Accession codes, unique identifiers, or web links for publicly available datasets
- A list of figures that have associated raw data
- A description of any restrictions on data availability

The single-cell RNA sequencing data have been deposited in GEO under accession number GSE124885 (https://www.ncbi.nlm.nih.gov/geo/query/acc.cgi?acc=GSE124885) and in NCBI Sequence Read Archive (SRA) under accession number PRJNA644060 (https://www.ncbi.nlm.nih.gov/sra/?term=PRJNA644060) on human and mouse γδ T cells, respectively. The datasets generated and analysed during the current study are available from the corresponding authors on reasonable request. The paired analysis of our data with existing scRNA-seq data titled “Single cell RNA sequencing of Vγ4 and Vγ6 γδT cells from different tissue”

under accession number GSE123400 (https://www.ncbi.nlm.nih.gov/geo/query/acc.cgi) was performed in this study. Source data are provided with this paper.

Field-specific reporting

Please select the one below that is the best fit for your research. If you are not sure, read the appropriate sections before making your selection.

[x] Life sciences   [ ] Behavioural & social sciences   [ ] Ecological, evolutionary & environmental sciences

For a reference copy of the document with all sections, see nature.com/documents/nr-reporting-summary-flat.pdf

Life sciences study design

All studies must disclose on these points even when the disclosure is negative.

Sample size	The in vitro culture assays described in this manuscript were performed 3-5 times. A minimum of 3 independent repeats is required to assess for a significant shift. The influenza infection, cell transfer, in vivo lipid antigen-treatment and in vivo antibody treatment data were performed on 2-4 different occasions using at least 3 mice per group, per standard protocols and to minimize the number of animals under experiment. Details regarding sample size of these and all other performed experiments are provided in the Methods section, the legends of the figures and in the supplementary files.
Data exclusions	All data generated during this study are shown in the manuscript.
Replication	All the reported experiments were reproducible. Data reproducibility was confirmed by two or three independent experiments.
Randomization	All strains of mice were randomly allocated into treated and control groups for the virus infection, cell transfer, in vivo lipid antigen-treatment and in vivo antibody-treatment experiments. Human participants were allocated into different groups based on the disease severity as provided in Supplementary Table 1 and in methods section.
Blinding	Groups of mice were blinded for collection and analysis of data when used for virus infection, cell transfer, in vivo lipid antigen-treatment and in vivo antibody-treatment studies. Sequencing was done blindly by Jingneng Co., Ltd. (Shanghai, China), which also provided the statistical analysis.

Reporting for specific materials, systems and methods

We require information from authors about some types of materials, experimental systems and methods used in many studies. Here, indicate whether each material, system or method listed is relevant to your study. If you are not sure if a list item applies to your research, read the appropriate section before selecting a response.

Materials & experimental systems

n/a	Involved in the study
[ ]	[x] Antibodies
[ ]	[x] Eukaryotic cell lines
[x]	[ ] Palaeontology
[ ]	[x] Animals and other organisms
[ ]	[x] Human research participants
[x]	[ ] Clinical data

Methods

n/a	Involved in the study
[x]	[ ] ChIP-seq
[ ]	[x] Flow cytometry
[x]	[ ] MRI-based neuroimaging

Antibodies

Antibodies used	All antibodies used in this study, including antibody specificity, supplier name and clone name are provided in the Methods section, the legends of the figures and in the supplementary files. For flow cytometry, the following antibodies from Biolegend were used: anti-mouse CD3 PE/Cy7 (clone 145-2C11), anti-mouse γδTCR Brilliant Violet 421™ (clone GL3), anti-mouse CD69 PerCP/Cy5.5 (clone H1.2F3), anti-mouse CD25 PE/Cy5 (clone PC61), anti-mouse NKG2D PE (clone CX5), anti-mouse NKG2A APC (clone 16A11), anti-mouse/human CD44 PE/Cy7 (clone IM7), anti-mouse PD-1 APC (clone 29F.1A12), anti-mouse CD62L PE/Cy5 (clone MEL-14), anti-mouse CD45 FITC (clone 30-F11), anti-mouse CD45.2 APC (clone 104), anti-mouse ICOS PE (clone 7E.17G9), anti-mouse CD4 PE/Cy5 (clone RM4-5), anti-mouse CD8 PerCP/Cy5.5 (clone 53-6.7), anti-mouse CD24 PE/Cy5 (clone M1/69), anti-mouse CD1d PerCP/Cy5.5 (clone 1B1), anti-mouse NK1.1 FITC (clone PK136), anti-mouse CD40 PE/Cy5 (clone 3/23), anti-mouse/rat/human CD27 PE/Cy7(clone LG.3A10), anti-mouse CXCR3 PE(clone CXCR3-173), anti-mouse CXCR4 PerCP/Cy5.5 (clone L276F12), anti-mouse CXCR5 PE (clone L138D7), anti-mouse/human CXCR7 PE/Cy7 (clone 8F11-M16), anti-mouse CCR6 APC (clone 29-2L17), anti-mouse CCR5 PE (clone HM-CCR5), anti-mouse CCR7 APC (clone 4B12), anti-mouse CD11a PE/Cy7 (clone M17/4), anti-mouse/human CD11b PE/Cy5 (clone M1/70), anti-mouse mouse CD11c PE/Cy7 (clone N418), anti-mouse CD49a PE (clone HMα1), anti-mouse CD49d PE (clone 9C10(MFR4.B)), anti-mouse CD29 FITC (clone HMβ1-1), anti-mouse CD9 FITC (clone MZ3), anti-human/mouse Integrin β7 FITC (clone FIB504), anti-mouse CD19 Brilliant Violet 421™ (clone 6D5), anti-mouse CD43 PE/Cy5 (clone 1B11), anti-mouse CD38 APC/Cy7 (clone 90), anti-mouse CD5 APC (clone 53-7.3), anti-mouse CD138 PE (clone 281-2), anti-mouse/human GL7 PerCP/Cy5.5 (clone GL7), anti-mouse IgG1 PE-CF594 (clone A85-1), anti-mouse IgM Brilliant Violet 421™ (clone RMM-1), anti-mouse Ly-6G/Ly-6C (Gr-1) FITC (clone RB6-8C5), anti-mouse Ly6G APC (clone 1A8), anti-mouse

	F4/80 PerCP/Cy5.5 (clone BM8), anti-mouse TER-119 FITC (clone Ly-76), anti-mouse TCR Vγ1.1 FITC (clone 2.11), anti-mouse TCR Vγ3 PE (clone 536), anti-mouse TCR Vγ4 APC (clone UC3-10A6), anti-rat IgM FITC (clone MRM-47), anti-human CD20 Brilliant Violet 421™ (clone 2H7), anti-human CD19 APC/Cy7 (clone HIB19), anti-mouse/rat/human CD27 PE/Cy7 (clone LG.3A10), anti-human CD43 APC (clone CD43-10G7), anti-human CD70 FITC (clone 113-16), anti-human CD1d PE (clone 51.1), antihuman CD3 PE/Cy7 (clone HIT3a), anti- human TCR γ/δ Brilliant Violet 421™ (clone B1), anti-human TCR Vγ9 APC (clone B3), anti-human IL-17A APC (clone BL168), anti-mouse IL-17A PE (clone TC11-18H10.1), anti-mouse Granzyme A PE (clone 3G8.5), anti-human/mouse Granzyme B (clone GB11), anti-mouse IL-4 PE (clone 11B11), anti-mouse IL-6 APC (clone MP5-20F3), anti-mouse IL-10 APC (clone JES5-16E3, BD Biosciences), anti-mouse IL-21 PE (clone mhalx21, eBioscience), anti-mouse IL-22 PE (clone Poly5164), anti-mouse IFN-γ PE (clone XMG1.2, eBioscience) and anti-human/mouse PE/Cy7 (clone 3E4, eBioscience).
Validation	All antibodies were obtained commercially were tested and validated by the respective company. All antibodies had validation statement provided on the website of the manufacturer.

Eukaryotic cell lines

Policy information about cell lines

Cell line source(s)	Madin-Darby Canine Kidney (MDCK) cell line from American Type Culture Collection (ATCC).
Authentication	The authentication was provided by ATCC.
Mycoplasma contamination	The cell line was tested negative for mycoplasma contamination by PCR.
Commonly misidentified lines (See ICLAC register)	Nil

Animals and other organisms

Policy information about studies involving animals; ARRIVE guidelines recommended for reporting animal research

Laboratory animals	Female mice between 6-8 weeks of age were used. IL-17A-deficient (Il17a−/−) mice on C57BL/6 background were obtained from Dr. Yoichiro Iwakura at The Institute of Medical Science, The University of Tokyo, Japan. IRF4-deficient (Irf4−/−) mice on C57BL/6 background were generated as previously described. C57BL/6 wild-type mice, B6.SJL-PtrcaPep3b/BoyJ (CD45.1) mice, C.Cg-Cd19tm1(cre)CgnIghb/J (Cd19−/−), Cd1d1f/f and Cd19Cre/+ mice were purchased from The Jackson Laboratory (Bar Harbor, ME, USA). All the mice were housed and bred under specific pathogen-free conditions at the animal facility of The University of Hong Kong, Shenzhen University School of Medicine, or Bioprocessing Technology Institute, Agency for Science, Technology and Research (A*STAR), Singapore. Mice were housed under specific pathogen-free conditions with a 12h light/dark cycle, at a temperature of 22 ± 2 °C, and a relative humidity of 50 ± 5%. Mice were fed a standard mouse chow diet.
Wild animals	Nil
Field-collected samples	Nil
Ethics oversight	All animal experiments were approved by Institutional Committee on the Use of Live Animals in Teaching and Research of The University of Hong Kong, Animal Ethical and Welfare Committee of Medical College at Shenzhen University, or A*STAR Biological Resource Centre Institutional Animal Care and Use Committee.

Note that full information on the approval of the study protocol must also be provided in the manuscript.

Human research participants

Policy information about studies involving human research participants

Population characteristics	The subjects of the study were hospitalized patients with CAP who underwent flexible fiberoptic bronchoscopy for investigation and treatment purposes in a respiratory clinic of a tertiary pediatric hospital in Guangzhou, China. Characteristics of the cohort was described in Table S1.
Recruitment	The participants of the study were hospitalized patients with CAP who underwent flexible fiberoptic bronchoscopy for investigation and treatment purposes in a respiratory clinic of a tertiary pediatric hospital in Guangzhou, China. No self-selection bias or other biases were present. Flexible bronchoscopy was performed for patients with CAP who met one or more of the following criteria: 1) the presence of lesions of unknown aetiology on the chest radiograph; 2) the need to evaluate recurrent pneumonia; 3) persistent pulmonary infiltrates or consolidation; 4) the need to investigate haemoptysis, persistent unexplained cough, dyspnoea, localized wheeze or stridor. Patients were prepared for bronchoscopy using inhalant lidocaine to minimize the cough reflex, as well as intravenous midazolam and atropine for moderate sedation and reduction of airway secretions. Bronchoalveolar lavage (BAL) was carried out in the most affected area identified radiologically and/or endoscopically. Warm sterile saline was injected at 2–3 mL/kg body weight for each affected lobe and recovered by aspiration into a suction trap under negative pressure of 6.65–13.3 kPa (50–100 mmHg). The BAL recovery rate was >40%. Severe CAP was defined according to a high-resolution CT scan (HRCT) score greater than or equal to 4, as well as clinical presentations that met the severe CAP criteria defined by the WHO (2005) 70 and the British Thoracic Society 71. Blood, throat swab, sputum and BAL samples were used for pathogen detection at the hospital diagnostic laboratories. Bacterial and fungal species were cultured in a Bactec9120 auto microbial culturing hood (BD) for 16–18 hours and characterized using a VITEK Compact System (Biomerieux-diagnostics Ltd.). Selected viral pathogens were detected by Taqman qPCR 72 and/or a Pneumoslide IgM ELISA kit. Assays were performed to detect Mastadenovirus (ADV), Bocavirus (BOV), Influenza virus A & B (FA, FB), Respiratory syncytial virus (RSV), Enterovirus (EV),

	Parainfluenza virus (PIV), Rhinovirus (RHV), Mycoplasma pneumoniae (MP) and Chlamydia pneumoniae (CP) (Vircell).
Ethics oversight	The study was approved by the Medical Ethics Committee of Guangzhou Women and Children's Medical Centre. Operations were performed according to the International Ethical Guidelines for Research Involving Human Subjects as stated in the Helsinki Declaration. The legal guardians of all participants provided informed written consents. Healthy PBMCs were isolated from buffy coat blood from healthy donors collected by the Hong Kong Red Cross with written consent. All experiments using PBMCs from healthy donors were approved by Institutional Review Board of The University of Hong Kong/ Hospital Authority Hong Kong West Cluster Institutional Review Board (HKU/ HA HKW IRB).

Note that full information on the approval of the study protocol must also be provided in the manuscript.

Flow Cytometry

Plots

Confirm that:

- [x] The axis labels state the marker and fluorochrome used (e.g. CD4-FITC).
- [x] The axis scales are clearly visible. Include numbers along axes only for bottom left plot of group (a 'group' is an analysis of identical markers).
- [x] All plots are contour plots with outliers or pseudocolor plots.
- [x] A numerical value for number of cells or percentage (with statistics) is provided.

Methodology

Sample preparation	Single-cell suspensions of mouse spleen, lymph node, thymus and lung were treated with red blood cell lysis buffer, then blocked with purified anti-mouse CD16/32 (clone 93, Biolegend) to reduce non-specific labeling, and the cells were thereafter stained with Live/Dead Zombie Aqua™ Fixable Viability dye (Biolegend) to stain for dead cells. Total lung lymphocytes were enriched by Percoll density centrifugation and collected from the interface of the 40 to 70% Percoll gradient.
Instrument	Samples were acquired on a BD FACSAria SORP (Becton Dickinson).
Software	Data were analyzed using FlowJo software (TreeStar).
Cell population abundance	The post-sort cells were checked for purity using a BD FACSAria SORP (Becton Dickinson, >98% purity) and then used for further analysis.
Gating strategy	The preliminary FSC/SSC gates of the starting cell population exclude debris and dead cells which have a lower level of forward scatter and are found at the bottom left corner of the contour plot. Doublet exclusion and live-cell discrimination was performed on all cell populations based on FSC-A/H. The cells were then stained with Live/Dead Zombie Aqua™ Fixable Viability dye (Biolegend) to stain for dead cells before further subgating (Figure exemplifies the gating strategy is provided in the Supplementary Figure 6a).

- [x] Tick this box to confirm that a figure exemplifying the gating strategy is provided in the Supplementary Information.
